# PCR-based detection and genetic characterization of porcine parvoviruses in South Korea in 2018

**DOI:** 10.1186/s12917-020-02329-z

**Published:** 2020-04-15

**Authors:** Hee-Chun Chung, Van-Giap Nguyen, Thi-My-Le Huynh, Yong-Ho Park, Kun-Taek Park, Bong-Kyun Park

**Affiliations:** 1 Department of Veterinary Medicine Virology Lab, College of Veterinary Medicine and Research Institute for Veterinary Science, Seoul National University DaeHakRo 1, GwanAk-Gu, Seoul, 151-742 Republic of Korea; 2 Department of Veterinary Microbiology and Infectious Diseases, Faculty of Veterinary Medicine, Vietnam National University of Agriculture, Hanoi, Vietnam; 3 Department of Veterinary Microbiology, College of Veterinary Medicine and Research Institute for Veterinary Science, Seoul National University, Seoul, 151-742 Republic of Korea; 4 Department of Biotechnology, Inje University, Gimhae, 50834 Republic of Korea

**Keywords:** Porcine parvoviruses, Detection, Genetic characterization, South Korea

## Abstract

**Background:**

with the advantage of sequencing technology, many novel porcine parvoviruses (PPV) rather than PPV1 has been reported. This study ultilized specific PCR- based method and gene- based analysis to study the presence and genetic diversity of porcine parvoviruses in South Korea in 2018.

**Results:**

The present study was conducted in 2018 and found PPV1 and PPV7 in nine out of 151 field samples (organs and semen) by the PCR method. Among these, the complete genome sequences of five strains (N2, N91, N108, N133, and N141) were recovered. Phylogenic analysis revealed that the strains N2, N91, and N108 belong to the PPV1 genotype, while N133 and N141 belong to PPV7 genotype. The PPV7 strains collected in this study had deletion mutations in the VP2 gene but differed from that of PPV7 strains collected in 2017. Among the PPV1 strains, the amino acid variations in the B cell epitopes of the VP2 protein were observed between three Korean PPV1 field strains (N2, N91, and N108) and the reference PPV1 strains. Those substitutions resulted in six out of 12 predicted epitopes having significant differences in antigenic index compared to the other PPV1 strains.

**Conclusions:**

This study confirmed the presence of different genotypes of porcine parvoviruses in South Korea. The PPVs circulating in South Korea were phylogenetically classified as PPV1 and PPV7 genotypes. Three Korean PPV1 strains collected in 2018 were predicted to have antigenic alteration in VP2 compared to several reference strains of PPV1.

## Background

Porcine parvoviruses (PPVs), which belong to the family *Parvoviridae*, subfamily *Parvovirinae*, are small, non-enveloped DNA viruses with a single-stranded linear genome of approximately 3.0–6.5 kb [1]. Over the past decade, several novel parvoviruses have been identified in pigs using molecular techniques, namely PPV2–PPV7. Unlike PPV1, which belongs to the genus *Protoparvovirus*, the emerging PPV species belong to the genera *Tetraparvovirus* (PPV2 and PPV3), *Copiparvovirus* (PPV4–PPV6), and unassigned genus *Chapparvovirus* (PPV7) [2, 3].

There are two open reading frames (ORF) presented in the porcine parvoviruses genome. One codes for large nonstructural proteins (NS1), which has low nucleotide substitution, and the other codes for structural viral proteins (VP1 and VP2) [2]. The two structural viral proteins VP1 and VP2 are considered related to the virulence of the virus that mainly targets the neutralization of antibodies against PPV1 [4–7].

Among seven genotypes of porcine parvoviruses, PPV1 is a well-known pathogen in pigs and is frequently associated with reproductive failure in swine. PPV1 usually causes fetal death in the absence of outward maternal clinical signs, thereby entailing the widespread vaccination of the breeding herd in an effort to control this virus [8, 9]. Although inactive vaccines are used in swine farms, PPV1 has not been eradicated and still poses several problems globally [10, 11].

To date, many studies have been conducted on the genotyping and topology of PPVs and analyzing their molecular genetics [2, 3, 9]. It has been suggested that PPVs are divided into 7 genotypes, of which genotype 7 was most recently identified. In South Korea, after the publications in 2001 [12] and 2017 [9, 13], few studies were focused on the detection of PPVs. This study attempted to investigate the presence of PPVs by molecular-based methods and investigate several genetic properties of PPVs.

## Results

### PCR-based detection of porcine parvoviruses

As shown in Table 1, PPVs were detected at a low rate of 5.96% (9/151 samples). Among the seven genotypes, only PPV1 and PPV7 were positive. PPV1 was discontinuously detected in January, April and May 2018. Seven PPV1- positive samples (fetuses and lungs) were collected from three provinces (Gyeongbuk, Chungbuk, and Chungnam). PPV7 was detected in two out of 15 semen samples collected from WH farm in Gyeongnam province.

**Table 1:** PCR- based detection of genotypes 1–7 of PPVs

Month	Number of samples	Number of PCR- positive samples						
		***PPV1***	***PPV2***	***PPV3***	***PPV4***	***PPV5***	***PPV6***	***PPV7***
January	16	4	0	0	0	0	0	0
February	48	0	0	0	0	0	0	0
March	26	0	0	0	0	0	0	0
April	16	1	0	0	0	0	0	0
May	9	2	0	0	0	0	0	0
June	6	0	0	0	0	0	0	0
July	8	0	0	0	0	0	0	0
August	22	0	0	0	0	0	0	2
**Total**	**151**	**7**	**0**	**0**	**0**	**0**	**0**	**2**

### Phylogenetic classification of porcine parvoviruses

**Fig. 1:**
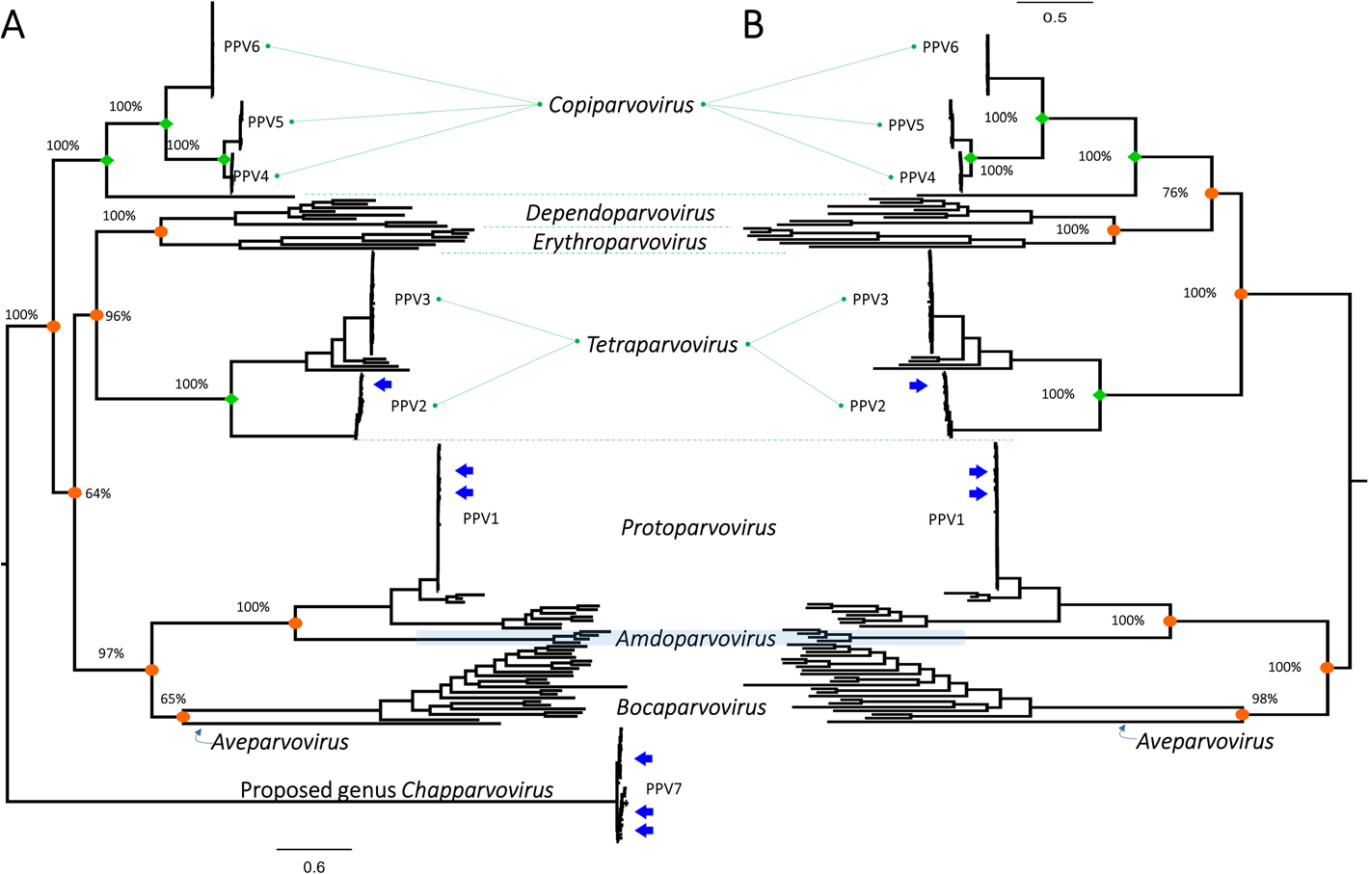
Maximum likelihood phylogenetic trees of porcine parvoviruses in the relationships with eight genera of subfamily *Parvovirinae*. The phylogenetic trees were inferred based on alignment of NS1 protein and were midpoint rooted. Given for the main internal nodes were the associated boostrap support values. The positions of Korean porcine parvoviruses in the phylogeny were indicated by arrows. The scale under each tree measures changes in the unit of the number of amino acid substitutions per site

Evaluated by the maximum likelihood mapping, the NS1 dataset (Additional file 1) was found to contain sufficient phylogenetic signal for tree inference as less than 20% of points distributed in the center of the triangle (Additional file 2). The patterns of phylogenetic clustering of PPV1- PPV7 genotypes were inferred in the relationship with eight recognized genera of subfamily *Parvovirinae*. Figure 1a and b consistently showed that (i) PPV4- PPV6 belonged to the genus *Copiparvovirus*, (ii) PPV2- PPV3 were within the genus *Tetraparvovirus*, and (iii) PPV1 was a member of the genus *Protoparvovirus*. Not grouping within any known genera of *Parvovirinae*, PPV7 clustered in the unassigned genus *Chapparvovirus* (Fig. 1a).

**Fig. 2:**
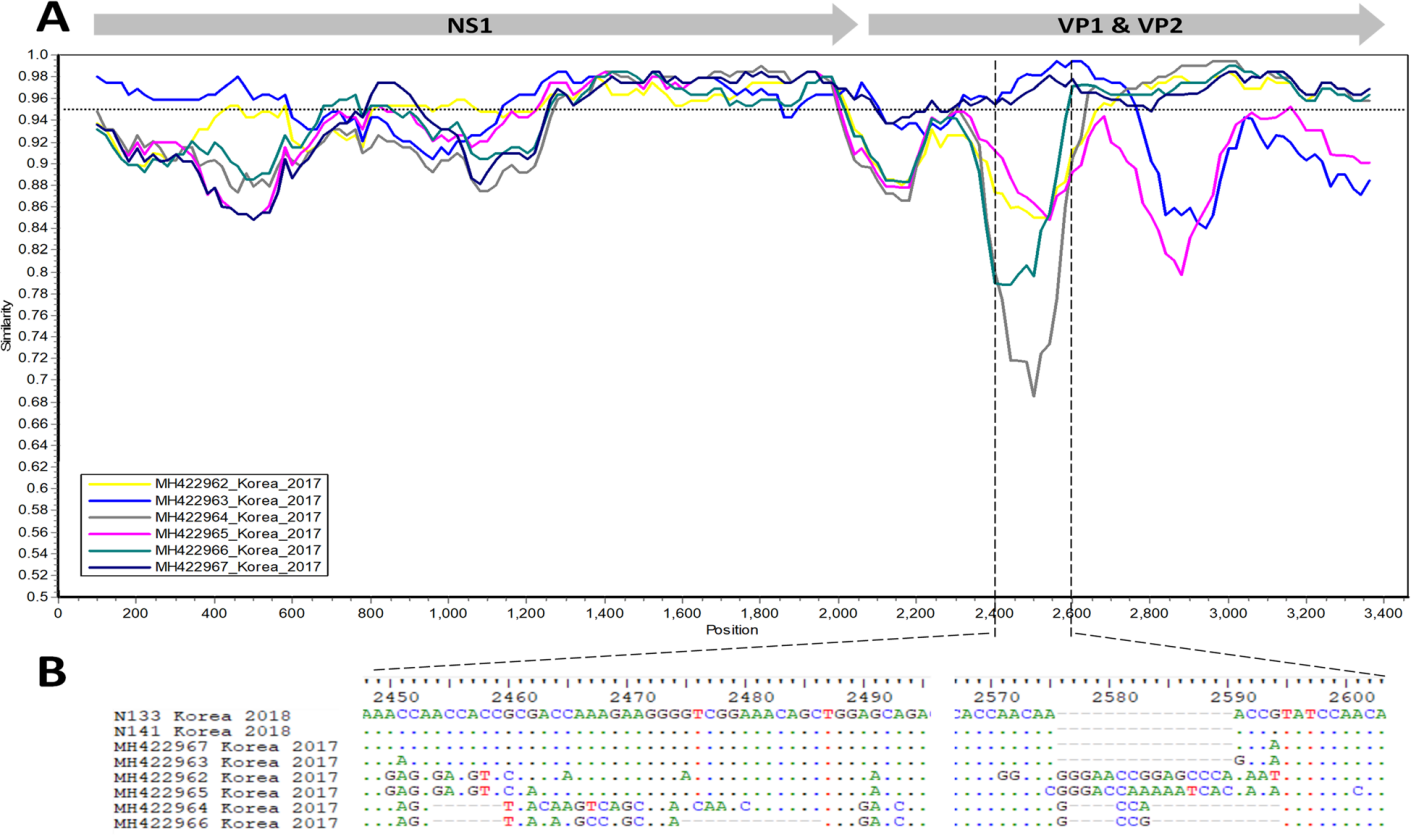
A comparison of the nucleotide sequences of Korean PPV7 strains. Panel **a** showed the sequence similarity between the two PPV7 strains (N133 and N141) collected in this study and six strains collected in 2017. The genomic region of VP2 gene that exhibits a drop of sequence similarity was indicated by a dashed line. The nucleotide comparison in panel **b** shows that the two PPV7 strains collected in 2018 had genetic deletion but were distinct from the six PPV7 strains sampled in 2017

Of the viruses circulating in South Korea collected in 2003 and 2016–2018 (arrows, Fig. 1a-b, Additional file 3), the phylogenetic trees reconstructed from three datasets showed that they were grouped within PPV1, PPV2, and PPV7 genotypes. Of these, the five sequences generated in this study were PPV1 (N2, N91, and N108) and PPV7 (N133 and N141). Focusing on the branching pattern of genotype 7 (Additional file 4), it was observed that Korean PPV7 strains scattered on different branches. This result might indicate the genetic diversity among PPV7s circulating in South Korea. The Simplot comparisons of the partial genome (3460 nucleotides) between two PPV7 strains generated in this study and six strains revealed several regions along the PPV7 genome that had < 95% nucleotide sequence similarity (dot line, Fig. 2a). In particular, the region between nucleotide 2400 and 2600 (limited by dashed lines, Fig. 2a) had a drop in sequence similarity. This region contained insertion and deletion mutations (Fig. 2b), of which two PPV7 strains (N133 and N141) had identical deletion compared to two strains collected in 2017 (MH422963 and MH422967).

### Evolutionary rates of porcine parvoviruses

**Fig. 3:**
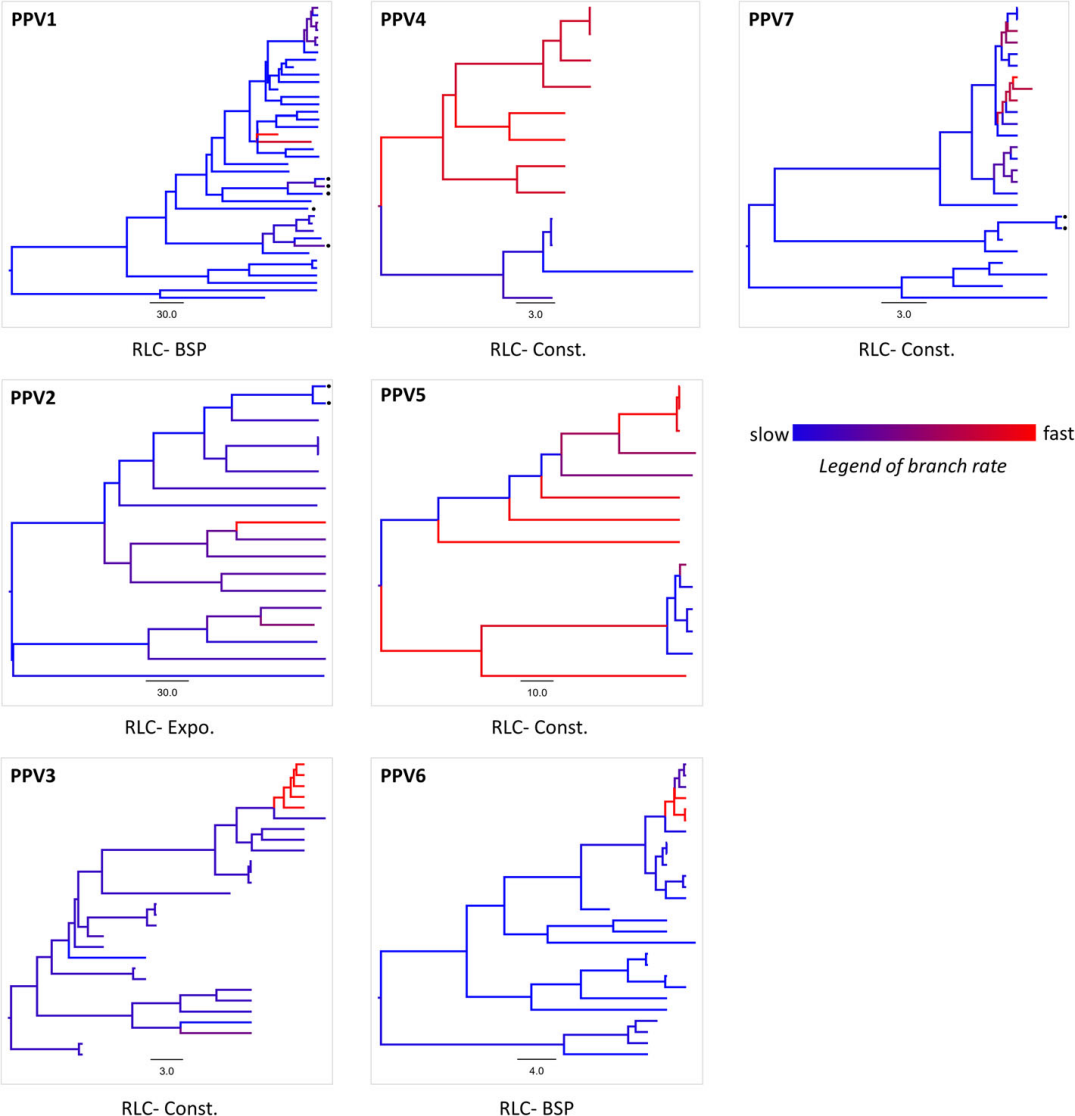
Branch rate heterogeneity observed in maximum clade credibility trees of PPV1- PPV7 genotypes based on genomic sequences. The trees were inferred from the data best-fit model of molecular clock (random local clock- RLC) and coalescent tree prior (Bayesian skyline plot- BSP; coalescent constant population- Const.; coalescent exponential population- Expo.). Branches are colored by inferred local clock rates (blue = slow, red = fast). Dots indicates the positions of Korean PPV strains in the phylogenetic tree. The time-scale (year) of evolutionary changes represented in the tree is indicated by the scale bar

Estimated separately for PPV1- PPV7 genotypes, the genome-wide rates were inferred from the models of best-fit molecular clock (Additional file 5) and coalescent tree prior (Additional file 6). In all genotypes, path sampling analyses supported the random local clock (RLC) as the best fit model since having lowest marginal likelihood (Additional file 5). The maximum clade credibility trees obtained from RLC model (Fig. 3) revealed rate heterogeneity across branches with mixture of slow and fast branch- specific rates.

**Fig. 4:**
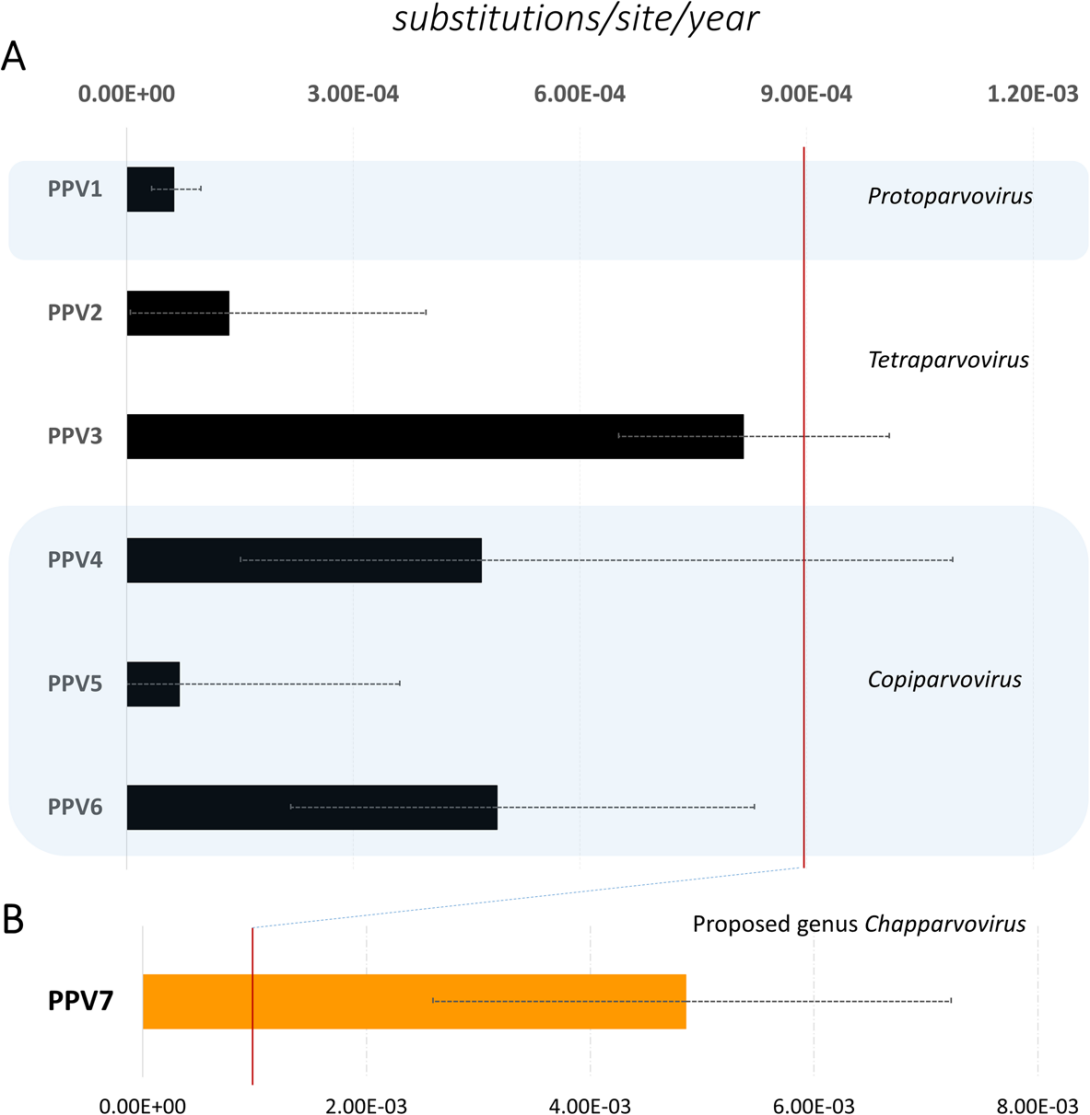
Estimated nucleotide substitution rates of complete genomes for each PPV1- PPV7 genotype. The genome-wide rates were inferred from the best-fit molecular clock and coalescent tree prior models. Error bars indicate 95% highest posterior density (HPD) interval. The vertical red line delimit rates less than one order of magnitude of 1 × 10^− 3^ subsitutions/ site/ year

The overall nucleotide substitution rates for 7 genotypes were depicted in Fig. 4. The genomes of PPV1- PPV6 were estimated to be evolving on the order of 10^− 5^ - 10^− 4^ nucleotide substitutions/site/year (Fig. 4a, Additional file 7). In a sharp contrast, PPV7 was estimated to be rapidly evolving on the order of 10^− 3^ nucleotide substitutions/site/year (Fig. 4b, Additional file 6). It was also observed that the substitution rates varied between genotypes of the same genus (PPV2 and PPV3 of genus *Tetraparvovirus*, and PPV4- PPV6 of genus *Copiparvovirus*, Fig. 4a).

### Pairwise genetic distances of porcine parvoviruses

**Fig. 5:**
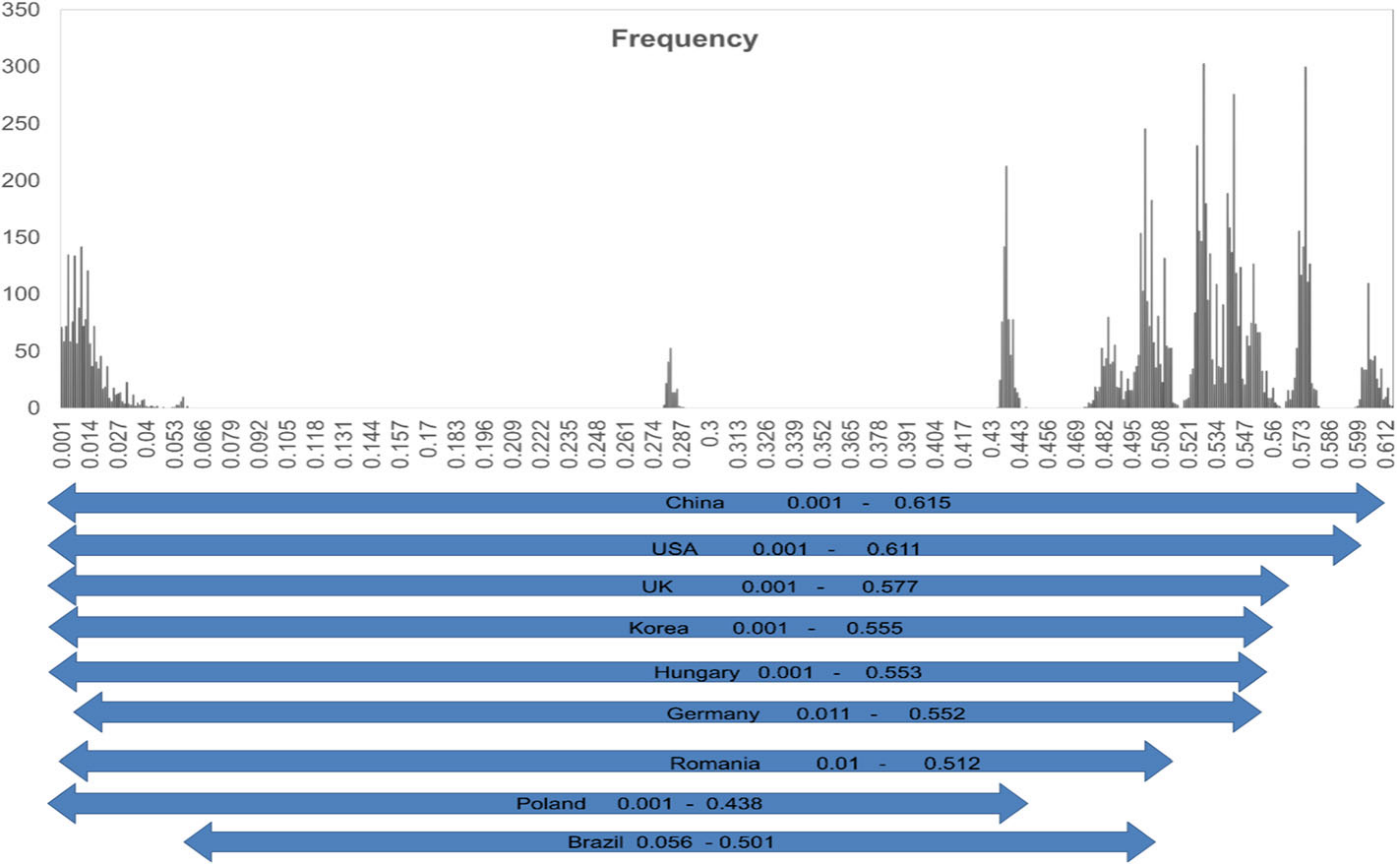
Comparison of the genetic distance between the seven PPV genotypes based on the complete genome. The frequency of each p-distance is shown as a column and the genetic distance of the virus in each country is indicated by arrows

The p-distance among 165 complete sequences of porcine parvoviruses (Additional file 8) were 0.001–0.615. Porcine parvoviruses detected in China showed the widest genetic variation (0.001–0.615). The genetic distance between the PPV strains in each country USA (0.001–0.611), UK (0.001–0.577), South Korea (0.001–0.555), Hungary (0.001–0.553), Germany (0.011–0.552), Romania (0.01–0.512), Poland (0.001–0.438), and Brazil (0.056–0.501) were almost within the range of that in China (Fig. 5). The results reflected the fact that there are multiple genotypes of PPV co-circulating in pigs in each country.

### Variations at the neutralizing epitope in the VP2 of three Korean PPV1 strains

**Fig. 6:**
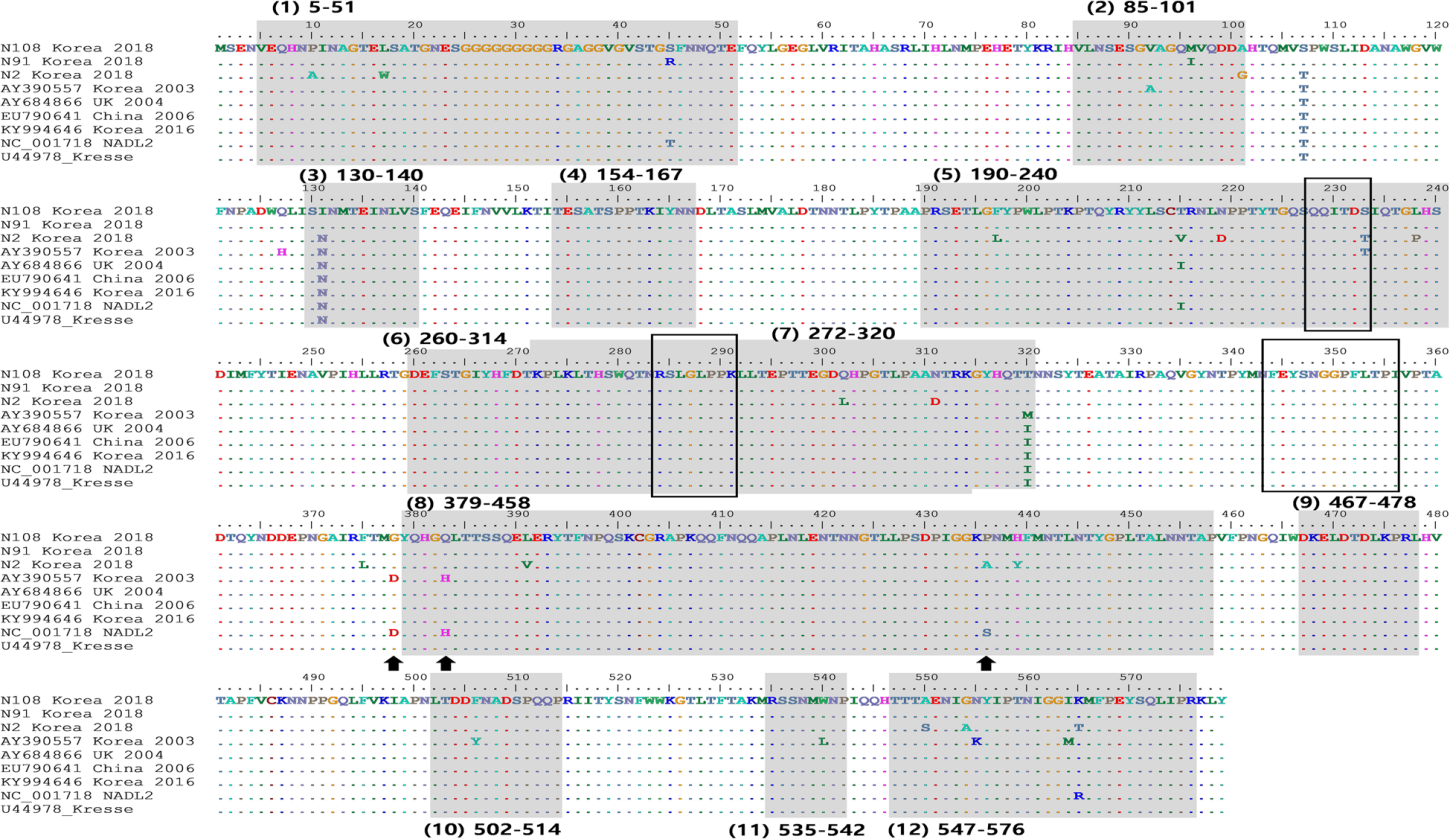
Amino acid alignment of the VP2 protein of PPV1. Twelve predicted B cell linear epitopes on the VP2 protein are shaded in gray. Three identified experimental linear B cell epitopes are marked as boxes. The amino acid substitutions that correspond to tissue tropism and attenuation are indicated by solid arrows

Using the B cell epitope prediction program, this study predicted 12 potential linear epitopes on the VP2 protein of PPV1: (1) 5–51, (2) 85–101, (3) 130–140, (4) 154–167, (5) 190–240, (6) 260–314, (7) 272–320, (8) 379–458, (9) 467–478, (10) 502–514, (11) 535–542, (12) 547–576. The amino acid variations in the B cell epitopes (Fig. 6) were observed between three Korean PPV1 field strains (N2, N91, and N108) and reference PPV1 strains. To access the influence of amino acid substitutions, the Jameson–Wolf antigenic index of VP2 were analyzed. As shown in Table 2, six out of the 12 predicted epitopes of the three Korean PPV1 field strains (N2, N91, and N108) showed significant differences in antigenic index with the other PPV1 strain (shade areas, Table 2). For example, epitope region (3) 130–140 had a negative and lower antigenic index than the other PPV1 in the comparison. The amino acid substitution N-131-I of N91 and N108 strains resulted in changes in the physical properties (hydrophilic–hydrophobic) of seven adjacent sites. The alteration of the antigenic index due to amino acid substitutions was also observed on epitope region 8 (sites 379–458) encompassing amino acid positions 378, 383, and 436, which responded to the tissue tropism of PPV1.

**Table 2:** Lists of amino acid sites of the VP2 protein that had alteration in the Jameson–Wolf antigenic index

Predicted Epitope	Position	U44978 Kresse	NC_001718 NADL2	KY994646 Korea 2016	EU790641 China 2006	AY684866 UK 2004	AY390557 Korea 2003	N2 Korea 2018	N91 Korea 2018	N108 Korea 2018
(1) 5–51	10	0.67	0.67	0.67	0.67	0.67	0.67	−0.1	0.67	0.67
(1) 5–51	11	0.88	0.88	0.88	0.88	0.88	0.88	− 0.4	0.88	0.88
(1) 5–51	12	1.29	1.29	1.29	1.29	1.29	1.29	0	1.29	1.29
(1) 5–51	42	−0.05	−0.05	− 0.05	− 0.05	− 0.05	−0.05	− 0.05	0.25	− 0.05
(1) 5–51	43	−0.05	− 0.05	−0.05	− 0.05	−0.05	− 0.05	−0.05	0.25	−0.05
(1) 5–51	51	0	0	0	0	0	0	0	0.6	0
(1) 5–51	103	0.26	0.26	0.26	0.26	0.26	0.26	0.53	−0.3	−0.1
(3) 130–140	130	0.48	0.48	0.48	0.48	0.48	0.48	0.48	−0.6	−0.6
(3) 130–140	131	0.87	0.87	0.87	0.87	0.87	0.87	0.87	−0.6	−0.6
(3) 130–140	132	2.16	2.16	2.16	2.16	2.16	2.16	2.16	−0.1	−0.1
(3) 130–140	133	2.4	2.4	2.4	2.4	2.4	2.4	2.4	−0.3	−0.3
(3) 130–140	134	1.96	1.96	1.96	1.96	1.96	1.76	1.96	−0.15	−0.15
(3) 130–140	135	0.77	0.77	0.77	0.77	0.77	0.77	0.77	−0.3	−0.3
(3) 130–140	136	−0.12	−0.12	− 0.12	−0.12	− 0.12	−0.12	− 0.12	−0.6	− 0.6
(5) 190–240	201	0.15	0.15	0.15	0.15	0.15	0.15	−0.05	0.15	0.15
(5) 190–240	235	−0.15	−0.15	− 0.15	−0.15	− 0.15	−0.15	0.66	−0.15	− 0.15
(5) 190–240	236	−0.25	− 0.25	−0.25	− 0.25	−0.25	− 0.45	0.17	− 0.25	−0.25
(5) 190–240	237	−0.05	−0.05	− 0.05	−0.05	− 0.05	−0.05	1.23	−0.05	− 0.05
(7) 272–320	317	−0.3	− 0.3	−0.3	− 0.3	−0.3	0.05	0.54	0.2	0.2
(7) 272–320	320	0	0	0	0	0	0.2	0.64	0.64	0.64
(7) 272–320	326	0	0	0	0	0	0	0.72	0.72	0.72
(8) 379–458	381	0.15	0.3	0.15	0.15	0.15	0.3	−0.05	0.15	0.15
(12) 547–576	567	0.25	0.25	0.25	0.25	0.25	0.25	−0.05	0.25	0.25
(12) 547–576	568	0.85	0.85	0.85	0.85	0.85	0.85	−0.05	0.85	0.85

## Discussion

Of the prevalence of porcine parvoviruses in South Korea, PPV1 is known as one of the most important causes of reproductive failure in swine. PPV1 has been known to cause economic losses in South Korea’s swine industry for more than 15 years [12]. However, few modern studies have focused on the presence of PPVs in general and PPV1 in particular. The low positive rate of PPV1 agreed with the previous studies where only one sample collected from 2013 to 2016 was positive for PPV1 [9]. Combined with two previous studies that detected PPV1 in South Korea [9, 12], it could be inferred that PPV1 distributed in several provinces, but at a low prevalence rate. The low PPV1 DNA detection rates in South Korea were in line with the situation described in certain European countries such as Poland [14] and Hungary [15].

Of the recently discovered PPV7, in combination with the first report of PPV7 in South Korea [13], this result confirmed again the presence of PPV7 in different provinces. In a previous study, PPV7 was found at a significantly higher rate from finishing pigs (74.9%) rather than aborted fetuses (24.0%) [13]. Additionally, it was reported that most PPV DNA-positive sera were identified in adult pigs aged 9–18 weeks [14]. Accordingly, the type of sample and age group of the pigs could affect to the detection rates for PPVs, which might be the main reason this study detected PPV7 at a very low rate of 1.32% compared to the previous study [13]. Genotypes 1–7 of PPV were detected in many countries, such as USA [16], Poland [14] and China [17], etc. However, to our best knowledge, only PPV1 [9, 12], PPV2 [18] and PPV7 [13] have been reported in South Korea to date. As a result, further study is required to elucidate the prevalence of the other PPV genotypes in this country.

To investigate the genetic diversity of porcine parvoviruses, this study reconstructed the genetic relationships of seven PPV genotypes with eight assigned genera of subfamily *Parvovirinae*. The clustering patterns of porcine parvoviruses in this study was in line with the previous publications of which PPV1- PPV7 belonged to different genera [13, 19–21]. The genetic heterogeneity of porcine parvoviruses was further revealed by the fact that the virus was not only evolving at high substitution rates (10^− 3^ to 10^− 5^ substitutions/site/year, Fig. 4) but also varying in rates of nucleotide substitution within each genus (Fig. 4). The high rate of viral evolution was previously known for some parvoviruses [22, 23].

On the VP2 capsid protein, three linear B cell epitopes were experimentally identified [6]. Following the previous publication [24], this study looked for substitutions in the VP2 capsid protein that are responsible for tissue tropism and pathogenicity. Compared to the non-pathogenic PPV1 strain (NADL2), none of Korean PPV1 (except AY390557, collected in 2003) had substitutions that corresponded to tissue tropism and attenuation (solid arrows, Fig. 4). Additionally, none of the Korean PPV1 strains had immune escape mutations (228-E and 419-Q) of the German 27a field isolate (AY684871) [25]. These results suggested that the three PPV1 field strains collected in this study were pathogenic. The VP2 protein of PPV1 encompassed major antigenic domains, which is regarded as a promising candidate immunogen with the capacity to induce the neutralization of antibodies [26]. It was hypothesized that the emergence of new capsid profiles could be due to viral adaptation to the broadly used PPV1 vaccines [27]. However, there is no available experiment data to date that validates the effect of amino acid substitutions on neutralizing epitopes. As a result, the antigenic alteration due to amino acid substitutions at a linear B cell epitope deserves further investigation.

## Conclusions

By the molecular-based method, this study confirmed the presence of different genotypes of porcine parvoviruses in South Korea. Of which, PPV1 was distributed in several provinces at a low prevalence rate. By genetic analysis, the PPVs circulating in South Korea were known to be within genotypes PPV1 and PPV7. Three Korean PPV1 strains collected in 2018 were predicted to have antigenic alteration in VP2 in compared to several reference strains of PPV1.

## Methods

### Sample collection and PCR-based detection of porcine parvoviruses

In January–August 2018, 151 samples (aborted fetuses, the lungs of dead sows, and the semen of boars) were randomly collected from 63 commercial farms in nine provinces (Additional file 9). All organs originated from dead pigs and they were requested for detection of PPVs from Boehringer Ingelheim Vetmedica Korea Ltd. (Grant no. 20180002). DNA was extracted from the pooled organs of the fetuses (heart, lung, spleen, and kidney), lungs, and semen according to the methods described previously [9]. The presence of PPV1–PPV7 was detected by PPV genotype specific primers: PPV1 [28], PPV2 and PPV3 [29], PPV4 and PPV5 [16], PPV6 [30], and PPV7 [3]. The PCR thermal profile was as follows: initial denaturation at 94 °C for 5 min, then 35 cycles of 94 °C for 30 s, 56 °C for 30 s, 72 °C for 45 s, and a final extension at 72 °C for 7 min.

### Complete genome sequencing of Korean porcine parvoviruses

For genetic characterization, five strains (N2, N91, N108, N133, and N141) were completely sequenced by the primer walking method. The strains N2, N91, and N108 utilized six pairs of overlapping primers [9], while the strains N133 and N141 utilized four pairs of overlapping primers [3]. The specific PCR products were purified by the gel extraction method and further processed for TA cloning and transformation [31]. The full-length genomes of N2, N91, N108, N133, and N141 strains were registered in GenBank (accession numbers: MH817779, MH817778, MH566237, MH817777, and MH817776).

### Data collection and sequence alignment

According to the previous publication [19], amino acid sequences of large nonstructural protein (NS1) were used to infer the genetic relationships between parvoviruses. Aimed at phylogenetic classification and ease of topology comparison, this study included reference sequences of eight recognized genera of the subfamily *Parvovirinae* (*Amdo-, Proto-, Ave-, Boca-, Copi-, Dependo-, Tetra-, and Erythroparvovirus*) [19]. The NS1 dataset contained (i) 59 reference sequences of known genus (ii) 165 sequences downloaded from GenBank, and (iii) five sequences of Korean parvoviruses generated in this study (Additional file 1). Because of high divergence, COBALT tool [32] was used to align NS1 sequences. That tool anchors the alignment using constraints derived from the conserved domain database (CDD) and PROSITE protein-motif database so that conserve residues were accurately aligned.

The evolutionary rates of each PPV1- PPV7 genotype were estimated from genomic sequences. The genomic collection of parvoviruses (*n* = 165, Additional file 8) were sampled from 1976 to 2018, originated from Asia (China, Korea, and Japan), America (USA and Brazil), and Europe (Poland, Hungary, United Kingdom, Germany, Romania, and Sweden). MAFFT [33] with default options was chosen to align genomic sequences.

### Phylogenetic analyses

Prior to phylogenetic reconstruction, the phylogenetic signal of NS1 dataset (Additional file 2) was evaluated by maximum likelihood mapping method [34] implemented in IQ-TREE program [35]. The dataset was not suitable for phylogenetic analysis if the percentage in the central of the triangle was more than 20–30% [36]. Using IQ-TREE v1.6.12 [35], the genetic relationships between parvoviruses were inferred by maximum likelihood method. The ‘-m MFP’ option was invoked which helps selecting the data best-fit amino acid substitution model. The branch support values were estimated by ultrafast bootstrap approximation [37] implemented in IQ-TREE [35] via “-bb 1000” option. The reconstructed phylogenies were displayed and midpoint rooted by FigTree v1.4.3 (http://tree.bio.ed.ac.uk/software/figtree/).

### Bayesian inference of evolutionary rates of porcine parvoviruses

The nucleotide substitution rates of each PPV1- PPV7 genotype were estimated based on genome alignments and used BEAST 2 package [38]. In these analyses, sequences without collection date were excluded. Details of PPV1- PPV7 datasets were given in Additional file 8. For model of nucleotide substitution, bModelTest tool [39] implemented in BEAST 2 was selected which helps to infer the most appropriate substitution model (Additional file 10). For molecular clock model, four models of strict clock, uncorrelated lognormal and exponential relaxed-clock [40], and random local clock [41] were specified. For tree prior, three coalescent models implemented in BEAST 2 were tested, including coalescent constant population, coalescent exponential population and coalescent Bayesian skyline plot [42]. In each analysis, two independent runs (100 million chains, sampling every 10,000 generations) were performed using BEAST package v2.6.1 [38], which is available at the CIPRES Science Gateway [43]. The output log files from multiple runs were combined using LogCombiner included in the BEAST package. The combined log files were subsequently analysed in Tracer v1.7.1 [44] to assess the convergence (effective sample size > 100). Interested in the evolutionary rates, this study subsequently performed path sampling analyses [45] to select the best fit molecular clock and tree prior models for each dataset. For that analysis, the number of path steps were 100, and the length of each chain were one million iterations. The nucleotide substitution rates of each PPV1- PPV7 genotype were only inferred from the data best-fit combining models (Additional files 5 and 6). The phylogenic trees were summarized with TreeAnnotator v2.6.1 to produce the maximum clade credibility tree, which was displayed using FigTree v1.4.3.

### Sliding window analysis for genetic variability

Nucleotide sequence similarity between two PPV7 strains collected in this study and six strains collected in 2017 [13] were analyzed by the Simplot program as described in a previous study [46]. Each plotted point is the percent genetic similarity within a 200-nucleotide wide sliding window centered on the position plotted with a step size of 20 nucleotides and Kimura 2-parameter.

### Calculation of the genetic distance

The genetic distance was calculated using the dataset of the complete genome sequence (*n* = 165, Additional file 8). The p-distance was estimated by MEGA 7 [47] and SDT v1.2 programs [48]. The genetic distance was represented by a frequency histogram.

### Variations in the neutralization of the epitope of Korean PPV1

Neutralizing epitopes on the VP2 protein of PPV1 and several epitopes were determined [6]. Additionally, the linear epitope of VP2 was predicted using BepiPred-2.0 version [49]. The Jameson–Wolf antigenic index implemented using Lasergene Protean software (DNASTAR, Inc., Madison, WI, USA) was used to predict whether amino acid substitutions would affect the antigenic properties of neutralizing epitopes. The antigenic index was calculated for each amino acid site and plotted using Microsoft Excel 2017 (Microsoft, Redmond, WA, USA).

## Supplementary information

**Additional file 1.** List of NS1 sequences.

**Additional file 2.** Maximum likelihood mapping.

**Additional file 3.** Phylogenetic tree of PPV1-PPV7.

**Additional file 4.** Phylogenetic tree of PPV7.

**Additional file 5.** Path sampling for clock model.

**Additional file 6.** Path sampling for coalescent model.

**Additional file 7.** Details of substitution rates.

**Additional file 8.** List of genomic sequences.

**Additional file 9.** Samples’ information.

**Additional file 10.** Posterior distribution of substitution models.

## Data Availability

All data generated or analyzed during this study are included in this published article and its additional files. The datasets analyzed in the current study are available on request from the corresponding author. The full-length genomes of N2, N91, N108, N133, and N141 strains were registered in GenBank with accession numbers: MH817779, MH817778, MH566237, MH817777, and MH817776.

## Abbreviations

PPV: Porcine parvoviruses
PPV1: Porcine parvovirus genotype 1
PPV2: Porcine parvovirus genotype 2
PPV3: Porcine parvovirus genotype 3
PPV4: Porcine parvovirus genotype 4
PPV5: Porcine parvovirus genotype 5
PPV6: Porcine parvovirus genotype 6
PPV7: Porcine parvovirus genotype 7
PCR: Polymerase chain reaction
ORF: Open reading frame
NS1: Large nonstructural proteins
VP gene: Structural viral protein-coding gene
UCLD: Uncorrelated lognormal relaxed- clock
UCED: Uncorrelated exponential relaxed- clock
RLC: Random local clock

## References

[1] Shackelton LA, Hoelzer K, Parrish CR, Holmes EC (2007). Comparative analysis reveals frequent recombination in the parvoviruses. J Gen Virol.

[2] Cadar D, Dan A, Tombacz K, Lorincz M, Kiss T, Becskei Z, Spinu M, Tuboly T, Csagola A (2012). Phylogeny and evolutionary genetics of porcine parvovirus in wild boars. Infect Genet Evol.

[3] Xing X, Zhou H, Tong L, Chen Y, Sun Y, Wang H, Zhang G (2018). First identification of porcine parvovirus 7 in China. Arch Virol.

[4] Xie HL, Wang Z, Cui SJ, Zhang CF, Cui YD (2010). The epitope of the VP1 protein of porcine parvovirus. Virol J.

[5] Sedlik C, Sarraseca J, Rueda P, Leclerc C, Casal I (1995). Immunogenicity of poliovirus B and T cell epitopes presented by hybrid porcine parvovirus particles. J Gen Virol.

[6] Sun J, Huang L, Wei Y, Wang Y, Chen D, Du W, Wu H, Feng L, Liu C (2015). Identification of three PPV1 VP2 protein-specific B cell linear epitopes using monoclonal antibodies against baculovirus-expressed recombinant VP2 protein. Appl Microbiol Biotechnol.

[7] Miyamura K, Kajigaya S, Momoeda M, Smith-Gill SJ, Young NS (1994). Parvovirus particles as platforms for protein presentation. Proc Natl Acad Sci.

[8] Kresse JI, Taylor WD, Stewart WW, Eernisse KA (1985). Parvovirus infection in pigs with necrotic and vesicle-like lesions. Vet Microbiol.

[9] Oh WT, Kim RY, Nguyen VG, Chung HC, Park BK (2017). Perspectives on the evolution of porcine parvovirus. Viruses.

[10] Martinez C, Dalsgaard K, Lopez de Turiso JA, Cortes E, Vela C, Casal JI (1992). Production of porcine parvovirus empty capsids with high immunogenic activity. Vaccine.

[11] Joo HS, Molitor TW, Leman AD (1984). Antibody responses of Guinea-pigs, rabbits and pigs to inactivated porcine parvovirus vaccines. Vet Microbiol.

[12] Lyoo KS, Park YH, Park BK (2001). Prevalence of porcine reproductive and respiratory syndrome virus, porcine circovirus type 2 and porcine parvovirus from aborted fetuses and pigs with respiratory problems in Korea. J Vet Sci.

[13] Ouh IO, Park S, Lee JY, Song JY, Cho IS, Kim HR, Park CK (2018). First detection and genetic characterization of porcine parvovirus 7 from Korean domestic pig farms. J Vet Sci.

[14] Cui J, Biernacka K, Fan J, Gerber PF, Stadejek T, Opriessnig T (2017). Circulation of porcine parvovirus types 1 through 6 in serum samples obtained from six commercial polish pig farms. Transbound Emerg Dis.

[15] Csagola A, Lorincz M, Cadar D, Tombacz K, Biksi I, Tuboly T (2012). Detection, prevalence and analysis of emerging porcine parvovirus infections. Arch Virol.

[16] Xiao CT, Gimenez-Lirola LG, Jiang YH, Halbur PG, Opriessnig T (2013). Characterization of a novel porcine parvovirus tentatively designated PPV5. PLoS One.

[17] Qin S, Ruan W, Yue H, Tang C, Zhou K, Zhang B (2018). Viral communities associated with porcine respiratory disease complex in intensive commercial farms in Sichuan province, China. Sci Rep.

[18] Lee JY, Kim EJ, Cho IS, Lee KK, Shin YK (2017). Complete genome sequences of porcine parvovirus 2 isolated from swine in the Republic of Korea. Genome Announc.

[19] Cotmore SF, Agbandje-McKenna M, Canuti M, Chiorini JA, Eis-Hubinger AM, Hughes J, Mietzsch M, Modha S, Ogliastro M, Penzes JJ (2019). ICTV virus taxonomy profile: Parvoviridae. J Gen Virol.

[20] Afolabi KO, Iweriebor BC, Okoh AI, Obi LC (2019). Increasing diversity of swine parvoviruses and their epidemiology in African pigs. Infect Genet Evol.

[21] Wang W, Cao L, Sun W, Xin J, Zheng M, Tian M, Lu H, Jin N (2019). Sequence and phylogenetic analysis of novel porcine parvovirus 7 isolates from pigs in Guangxi, China. PLoS One.

[22] Shackelton LA, Parrish CR, Truyen U, Holmes EC (2005). High rate of viral evolution associated with the emergence of carnivore parvovirus. Proc Natl Acad Sci U S A.

[23] Fan W, Sun Z, Shen T, Xu D, Huang K, Zhou J, Song S, Yan L (2017). Analysis of evolutionary processes of species jump in waterfowl parvovirus. Front Microbiol.

[24] Bergeron J, Hebert B, Tijssen P (1996). Genome organization of the Kresse strain of porcine parvovirus: identification of the allotropic determinant and comparison with those of NADL-2 and field isolates. J Virol.

[25] Zeeuw EJ, Leinecker N, Herwig V, Selbitz HJ, Truyen U (2007). Study of the virulence and cross-neutralization capability of recent porcine parvovirus field isolates and vaccine viruses in experimentally infected pregnant gilts. J Gen Virol.

[26] Molitor TW, Joo HS, Collett MS (1983). Porcine parvovirus: virus purification and structural and antigenic properties of virion polypeptides. J Virol.

[27] Streck AF, Canal CW, Truyen U (2015). Molecular epidemiology and evolution of porcine parvoviruses. Infect Genet Evol.

[28] Jiang Y, Shang H, Xu H, Zhu L, Chen W, Zhao L, Fang L (2010). Simultaneous detection of porcine circovirus type 2, classical swine fever virus, porcine parvovirus and porcine reproductive and respiratory syndrome virus in pigs by multiplex polymerase chain reaction. Vet J.

[29] Streck AF, Homeier T, Foerster T, Fischer S, Truyen U (2013). Analysis of porcine parvoviruses in tonsils and hearts from healthy pigs reveals high prevalence and genetic diversity in Germany. Arch Virol.

[30] Schirtzinger EE, Suddith AW, Hause BM, Hesse RA (2015). First identification of porcine parvovirus 6 in North America by viral metagenomic sequencing of serum from pigs infected with porcine reproductive and respiratory syndrome virus. Virol J.

[31] Kim AR, Chung HC, Kim HK, Kim EO, Nguyen VG, Choi MG, Yang HJ, Kim JA, Park BK (2014). Characterization of a complete genome of a circular single-stranded DNA virus from porcine stools in Korea. Virus Genes.

[32] Papadopoulos JS, Agarwala R (2007). COBALT: constraint-based alignment tool for multiple protein sequences. Bioinformatics.

[33] Katoh K, Standley DM (2013). MAFFT multiple sequence alignment software version 7: improvements in performance and usability. Mol Biol Evol.

[34] Strimmer K, von Haeseler A (1997). Likelihood-mapping: a simple method to visualize phylogenetic content of a sequence alignment. Proc Natl Acad Sci U S A.

[35] Nguyen L-T, Schmidt HA, von Haeseler A, Minh BQ (2015). IQ-TREE: a fast and effective stochastic algorithm for estimating maximum-likelihood phylogenies. Mol Biol Evol.

[36] Schmidt HA, von Haeseler A, Vandamme A-M, Salemi M, Lemey P (2009). Phylogenetic inference using maximum likelihood methods. The Phylogenetic Handbook: A Practical Approach to Phylogenetic Analysis and Hypothesis Testing.

[37] Hoang DT, Chernomor O, von Haeseler A, Minh BQ, Vinh LS (2017). UFBoot2: improving the ultrafast bootstrap approximation. Mol Biol Evol.

[38] Bouckaert R, Vaughan TG, Barido-Sottani J, Duchene S, Fourment M, Gavryushkina A, Heled J, Jones G, Kuhnert D, De Maio N (2019). BEAST 2.5: An advanced software platform for Bayesian evolutionary analysis. PLoS Comput Biol.

[39] Bouckaert RR, Drummond AJ (2017). bModelTest: Bayesian phylogenetic site model averaging and model comparison. BMC Evol Biol.

[40] Drummond AJ, Ho SY, Phillips MJ, Rambaut A (2006). Relaxed phylogenetics and dating with confidence. PLoS Biol.

[41] Drummond AJ, Suchard MA (2010). Bayesian random local clocks, or one rate to rule them all. BMC Biol.

[42] Drummond AJ, Rambaut A, Shapiro B, Pybus OG (2005). Bayesian coalescent inference of past population dynamics from molecular sequences. Mol Biol Evol.

[43] Miller MA, Pfeiffer W, Schwartz T (2010). Creating the CIPRES Science Gateway for inference of large phylogenetic trees. 2010 Gateway Computing Environments Workshop (GCE): 14–14 Nov. 2010 2010.

[44] Rambaut A, Drummond AJ, Xie D, Baele G, Suchard MA (2018). Posterior summarization in Bayesian Phylogenetics using tracer 1.7. Syst Biol.

[45] Baele G, Lemey P, Bedford T, Rambaut A, Suchard MA, Alekseyenko AV (2012). Improving the accuracy of demographic and molecular clock model comparison while accommodating phylogenetic uncertainty. Mol Biol Evol.

[46] Lole KS, Bollinger RC, Paranjape RS, Gadkari D, Kulkarni SS, Novak NG, Ingersoll R, Sheppard HW, Ray SC (1999). Full-length human immunodeficiency virus type 1 genomes from subtype C-infected seroconverters in India, with evidence of intersubtype recombination. J Virol.

[47] Kumar S, Stecher G, Tamura K (2016). MEGA7: molecular evolutionary genetics analysis version 7.0 for bigger datasets. Mol Biol Evol.

[48] Muhire BM, Varsani A, Martin DP (2014). SDT: a virus classification tool based on pairwise sequence alignment and identity calculation. PLoS One.

[49] Jespersen MC, Peters B, Nielsen M, Marcatili P (2017). BepiPred-2.0: improving sequence-based B-cell epitope prediction using conformational epitopes. Nucleic Acids Res.

