# Supplementary material for: PCR-based detection and genetic characterization of porcine parvoviruses in South Korea in 2018
Source: BMC Vet Res. 2020 Apr 15;16:113. doi: 10.1186/s12917-020-02329-z (PMC7161289; doi:10.1186/s12917-020-02329-z)
Supplement: Supplementary file 1 — Additional file 1. List of NS1 sequences. [file 12917_2020_2329_MOESM1_ESM.docx]

List of the 229 NS1 sequences used for phylogenetic analysis.

| **No.** | **Name of sequences** | **Type** |
| --- | --- | --- |
| 1 | GU938300_porcine_parvovirus_2_Tetraparvovirus | References |
| 2 | JQ037753_Eidolon_helvum_(bat)_parvovirus_1_Tetraparvovirus | References |
| 3 | AY622943_human_parvovirus_4_Tetraparvovirus | References |
| 4 | EU200677_porcine_hokovirus;_porcine_parvovirus_3_Tetraparvovirus | References |
| 5 | JF504699_ovine_hokovirus_1_Tetraparvovirus | References |
| 6 | EU200669_bovine_hokovirus_1_Tetraparvovirus | References |
| 7 | GQ200736_chipmunk_parvovirus_Erythroparvovirus | References |
| 8 | AF406967_bovine_parvovirus_3_Erythroparvovirus | References |
| 9 | AY386330_human_parvovirus_B19_Erythroparvovirus | References |
| 10 | AF221123_pig-tailed_macaque_parvovirus_Erythroparvovirus | References |
| 11 | AF221122_rhesus_macaque_parvovirus_Erythroparvovirus | References |
| 12 | U26342_simian_parvovirus_Erythroparvovirus | References |
| 13 | AY349010_snake_adeno-associated_virus_Dependoparvovirus | References |
| 14 | KP733794_Bearded_dragon_parvovirus_Dependoparvovirus | References |
| 15 | U22967_Muscovy_duck_parvovirus_Dependoparvovirus | References |
| 16 | GU226971_bat_adeno-associated_virus_Dependoparvovirus | References |
| 17 | AY186198_avian_adeno-associated_virus_Dependoparvovirus | References |
| 18 | AF043303_adeno-associated_virus_2_Dependoparvovirus | References |
| 19 | JN420372_Californian_sea_lion_adeno-associated_virus_1_Dependoparvovirus | References |
| 20 | AF085716_adeno-associated_virus_5_Dependoparvovirus | References |
| 21 | AF406966_bovine_parvovirus_2_Copiparvovirus | References |
| 22 | GQ387499_porcine_parvovirus_4_Copiparvovirus | References |
| 23 | GU214706_turkey_parvovirus_Aveparvovirus | References |
| 24 | DQ335247_bovine_parvovirus_1_Bocaparvovirus | References |
| 25 | KU172421_bovine_bocaparvovirus_2_Bocaparvovirus | References |
| 26 | JF429834_porcine_bocavirus_3_Bocaparvovirus | References |
| 27 | JQ814850_Myotis_myotis_(bat)_bocavirus_1_Bocaparvovirus | References |
| 28 | FJ170278_human_bocavirus_2c_Bocaparvovirus | References |
| 29 | JQ923422_human_bocavirus_1_Bocaparvovirus | References |
| 30 | KU321654_Miniopterus_schreibersii_bat_bocavirus_Bocaparvovirus | References |
| 31 | KP729195_rabbit_bocaparvovirus_Bocaparvovirus | References |
| 32 | HQ223038_porcine_bocavirus_SX_Bocaparvovirus | References |
| 33 | HQ291308_porcine_bocavirus_H18_Bocaparvovirus | References |
| 34 | HM053693_porcine_bocavirus_1_Bocaparvovirus | References |
| 35 | KC339251_bat_bocavirus_WM40_Bocaparvovirus | References |
| 36 | KC339250_bat_bocavirus_XM30_Bocaparvovirus | References |
| 37 | KU950356_mink_bocavirus_1_Bocaparvovirus | References |
| 38 | KF792837_feline_bocaparvovirus_2_Bocaparvovirus | References |
| 39 | KM017744_feline_bocaparvovirus_3_Bocaparvovirus | References |
| 40 | JQ692585_feline_bocavirus_Bocaparvovirus | References |
| 41 | FJ214110_minute_virus_of_canines_Bocaparvovirus | References |
| 42 | JN420361_Californian_sea_lion_bocavirus_1_Bocaparvovirus | References |
| 43 | JN648103_canine_bocavirus_1_Bocaparvovirus | References |
| 44 | JN420365_Californian_sea_lion_bocavirus_3_Bocaparvovirus | References |
| 45 | JN202450_gray_fox_amdovirus_Amdoparvovirus | References |
| 46 | KJ396347_racoon_dog_and_fox_amdoparovirus_Amdoparvovirus | References |
| 47 | KX981923_skunk_amdoparovirus_Amdoparvovirus | References |
| 48 | M20036_Aleutian_mink_disease_parvovirus_Amdoparvovirus | References |
| 49 | L23427_porcine_parvovirus_Protoparvovirus | References |
| 50 | M19296_canine_parvovirus_Protoparvovirus | References |
| 51 | J02275_minute_virus_of_mice_Protoparvovirus | References |
| 52 | AF036710_rat_parvovirus_1_Protoparvovirus | References |
| 53 | LC085675_megabat_bufavirus_1_Protoparvovirus | References |
| 54 | JX027296_bufavirus_1a_(human)_Protoparvovirus | References |
| 55 | KT868811 cutavirus_(human)_Protoparvovirus | References |
| 56 | AB937988_Mpulungu_(shrew)_bufavirus_Protoparvovirus | References |
| 57 | KT716186_rat_bufavirus_SY-2015_Protoparvovirus | References |
| 58 | JX627576_Wuharv_(rhesus)_parvovirus_1_Protoparvovirus | References |
| 59 | KT965075_porcine_bufavirus_Protoparvovirus | References |
| 60 | KF429254_HN-I_China_PPV1_2013 | PPV1 |
| 61 | KX242359_GD2013_China_PPV1_2013 | PPV1 |
| 62 | KF429255_HN-K_China_PPV1_2013 | PPV1 |
| 63 | AY583318_China_China_PPV1_2013 | PPV1 |
| 64 | JN968975_JT_China_PPV1_2010 | PPV1 |
| 65 | M38367_NADL-2_UK_PPV1_1990 | PPV1 |
| 66 | NC_001718_NADL-2_USA_PPV1_1976 | PPV1 |
| 67 | KF913349_NADL-2 M13_Hungary_PPV1_2012 | PPV1 |
| 68 | KF913347_NADL-2 M3_Hungary_PPV1_2012 | PPV1 |
| 69 | KF913350_NADL-2 M23_Hungary_PPV1_2012 | PPV1 |
| 70 | KF913351_NADL-2 M123_Hungary_PPV1_2012 | PPV1 |
| 71 | KF913348_NADL-2 M12_Hungary_PPV1_2012 | PPV1 |
| 72 | KF913346_NADL-2 M2_Hungary_PPV1_2012 | PPV1 |
| 73 | KF913345_NADL-2 M1_Hungary_PPV1_2012 | PPV1 |
| 74 | KF742500_J-PPV_China_PPV1_2013 | PPV1 |
| 75 | KF429252_HN-G_China_PPV1_2012 | PPV1 |
| 76 | DQ675456_SR-1_China_PPV1_2006 | PPV1 |
| 77 | KF429253_HN-H_China_PPV1_2012 | PPV1 |
| 78 | D00623_NADL-2_USA_PPV1_1976 | PPV1 |
| 79 | JX992846_HN-2011_China_PPV1_2008 | PPV1 |
| 80 | MF447833_HNLY201301_China_PPV1_2013 | PPV1 |
| 81 | U44978_Kresse_USA_PPV1_1985 | PPV1 |
| 82 | AY684866_Challenge_Germany_PPV1_1986 | PPV1 |
| 83 | KY994646_T142_Korea_PPV1_2016 | PPV1 |
| 84 | EU790641_BQ_China_PPV1_2006 | PPV1 |
| 85 | AY390557_VRI-1_Korea_PPV1_2003 | PPV1 |
| 86 | JN400516_7a_Germany_PPV1_2011 | PPV1 |
| 87 | JN400517_8a_Germany_PPV1_2011 | PPV1 |
| 88 | AY684869_Tornau/1/02_Germany_PPV1_2011 | PPV1 |
| 89 | AY684872_vaccine virus IDT_Germany_PPV1_1964 | PPV1 |
| 90 | JN400518_14a_Germany_PPV1_2011 | PPV1 |
| 91 | JN400519_693a_Germany_PPV1_2011 | PPV1 |
| 92 | KJ201927_HNAY_China_PPV1_2009 | PPV1 |
| 93 | KJ201928_HNZK-1_China_PPV1_2007 | PPV1 |
| 94 | FJ822038_Nanjing200801_China_PPV1_2008 | PPV1 |
| 95 | KX233726_TJ_China_PPV1_2015 | PPV1 |
| **96** | **MH566237_N108_Korea_PPV1_2018** | **PPV1** |
| **97** | **MH817778_N91_Korea_PPV1_2018** | **PPV1** |
| **98** | **MH817779_N2_Korea_PPV1_2018** | **PPV1** |
| 99 | EU790642_ZJ_China_PPV1_2004 | PPV1 |
| 100 | KY018935_GBGW1_Korea_PPV2_2016 | PPV2 |
| 101 | KY018936_GBGW2_Korea_PPV2_2016 | PPV2 |
| 102 | JX101461_US-135_USA_PPV2_2011 | PPV2 |
| 103 | KM926355_BR/GO/ion_09_Brazil_PPV2_2011 | PPV2 |
| 104 | NC_025965_BR/GO/ion_09_Brazil_PPV2_2011 | PPV2 |
| 105 | JX101462_US-523_USA_PPV2_2011 | PPV2 |
| 106 | MG345014_S4_China_PPV2_2017 | PPV2 |
| 107 | AB916464_JPT68_Japan_PPV2_2018 | PPV2 |
| 108 | MG345017_S18_China_PPV2_2017 | PPV2 |
| 109 | MG345016_S16_China_PPV2_2017 | PPV2 |
| 110 | MG345015_S9_China_PPV2_2017 | PPV2 |
| 111 | MG345013_S1_China_PPV2_2017 | PPV2 |
| 112 | MG345018_S23_China_PPV2_2017 | PPV2 |
| 113 | KU745627_GX_China_PPV2_2015 | PPV2 |
| 114 | KP765690_PPV2_Hungary_PPV2_2013 | PPV2 |
| 115 | KY586144_PPV2_Brazil_PPV2_2008 | PPV2 |
| 116 | KC296749_98T_Germany_PPV2_2010 | PPV2 |
| 117 | MG345019_SERUM-SMU_China_PPV2_2017 | PPV2 |
| 118 | JQ868701_F5-BH_Romania_PPV3_2010 | PPV3 |
| 119 | JQ868703_F6-4BV_Romania_PPV3_2010 | PPV3 |
| 120 | JQ868699_F7-1AB_Romania_PPV3_2010 | PPV3 |
| 121 | JQ868705_F1-10M_Romania_PPV3_2010 | PPV3 |
| 122 | JQ868706_F2-47M_Romania_PPV3_2010 | PPV3 |
| 123 | FJ982249_F8-1994A_UK_PPV3_1994 | PPV3 |
| 124 | FJ982250_F8-1994B_UK_PPV3_1994 | PPV3 |
| 125 | FJ982253_F8-1999_UK_PPV3_1999 | PPV3 |
| 126 | FJ982255_F8-2000B_UK_PPV3_2000 | PPV3 |
| 127 | FJ982254_F8-2000A_UK_PPV3_2000 | PPV3 |
| 128 | FJ982251_F8-1996A_UK_PPV3_1996 | PPV3 |
| 129 | FJ982247_Cl2001B_UK_PPV3_2001 | PPV3 |
| 130 | FJ982248_Cl2001C_UK_PPV3_2001 | PPV3 |
| 131 | FJ982246_Cl2001A_UK_PPV3_2001 | PPV3 |
| 132 | FJ982252_F8-1996B_UK_PPV3_1996 | PPV3 |
| 133 | KU167029_GX2_China_PPV3_2015 | PPV3 |
| 134 | KX827774_YL18_China_PPV3_2015 | PPV3 |
| 135 | KU167028_GX1_China_PPV3_2015 | PPV3 |
| 136 | KX827776_BB168_China_PPV3_2015 | PPV3 |
| 137 | KX827777_YL173_China_PPV3_2015 | PPV3 |
| 138 | MG345026_SERUM-SMU_China_PPV3_2017 | PPV3 |
| 139 | KX827772_NN200_China_PPV3_2015 | PPV3 |
| 140 | KX827773_YL172_China_PPV3_2015 | PPV3 |
| 141 | KX827775_DX101_China_PPV3_2015 | PPV3 |
| 142 | JQ868702_F3-3NB_Romania_PPV3_2010 | PPV3 |
| 143 | JQ868704_F4-7SM_Romania_PPV3_2010 | PPV3 |
| 144 | JQ868700_F9-1MS_Romania_PPV3_2010 | PPV3 |
| 145 | KY586145_parvovirus 3_Brazil_PPV3_2008 | PPV3 |
| 146 | NC_014665_17_USA_PPV4_2006 | PPV4 |
| 147 | MG345027_SERUM-SMU_China_PPV4_2017 | PPV4 |
| 148 | GQ387500_14_USA_PPV4_2006 | PPV4 |
| 149 | JQ868715_WB-542BH_Romania_PPV4_2007 | PPV4 |
| 150 | JQ868716_WB-549BH_Romania_PPV4_2007 | PPV4 |
| 151 | HM031134_JS0918a_China_PPV4_2009 | PPV4 |
| 152 | HM031135_JS0918b_China_PPV4_2009 | PPV4 |
| 153 | GU978967_JS0910-5644_China_PPV4_2009 | PPV4 |
| 154 | GU978965_HEN0922-5645_China_PPV4_2009 | PPV4 |
| 155 | JQ868713_WB-195HR_Romania_PPV4_2007 | PPV4 |
| 156 | JQ868714_WB-209CV_Romania_PPV4_2007 | PPV4 |
| 157 | KX352455_PPV5-P13-9_Poland_PPV5_2016 | PPV5 |
| 158 | KX352456_PPV5-P13-10_Poland_PPV5_2016 | PPV5 |
| 159 | KX352457_PPV5-P12-1_Poland_PPV5_2016 | PPV5 |
| 160 | KX273436_K17-4_Poland_PPV5_2013 | PPV5 |
| 161 | KX352458_PPV5-K17-1_Poland_PPV5_2016 | PPV5 |
| 162 | KF661535_HN01_China_PPV5_2013 | PPV5 |
| 163 | JX896322_ND564_USA_PPV5_2011 | PPV5 |
| 164 | JX896319_IN273_USA_PPV5_2011 | PPV5 |
| 165 | JX896318_MI216_USA_PPV5_2011 | PPV5 |
| 166 | KU745628_GX_China_PPV5_2015 | PPV5 |
| 167 | JX896321_IA469_USA_PPV5_2011 | PPV5 |
| 168 | NC_023020_IA469_USA_PPV5_2011 | PPV5 |
| 169 | JX896320_IA469_USA_PPV5_2011 | PPV5 |
| 170 | MG345028_SERUM SMU_China_PPV5_2017 | PPV5 |
| 171 | KX384815_U18-7_Poland_PPV6_2016 | PPV6 |
| 172 | KX384816_U18-8_Poland_PPV6_2016 | PPV6 |
| 173 | KX384817_U18-9_Poland_PPV6_2016 | PPV6 |
| 174 | KX384822_U18-1_Poland_PPV6_2016 | PPV6 |
| 175 | KX384814_U18-4_Poland_PPV6_2016 | PPV6 |
| 176 | KX384818_U18-5_Poland_PPV6_2016 | PPV6 |
| 177 | KX384819_K13-4_Poland_PPV6_2016 | PPV6 |
| 178 | KR709265_KSU4-NE-2014_USA_PPV6_2014 | PPV6 |
| 179 | KR709266_KSU5-NE-2014_USA_PPV6_2014 | PPV6 |
| 180 | KR709268_KSU7-SD-2014_USA_PPV6_2014 | PPV6 |
| 181 | KX384820_K17-10_Poland_PPV6_2016 | PPV6 |
| 182 | KX384821_K17-3_Poland_PPV6_2016 | PPV6 |
| 183 | KX384813_K13-8_Poland_PPV6_2016 | PPV6 |
| 184 | KY094494_Br_Brazil_PPV6_2008 | PPV6 |
| 185 | KR709262_KSU1-AZ-2014_USA_PPV6_2014 | PPV6 |
| 186 | KR709264_KSU3-KS-2014_USA_PPV6_2014 | PPV6 |
| 187 | MG345036_SERUM-SMU_China_PPV6_2017 | PPV6 |
| 188 | KF999685_TJ_China_PPV6_2012 | PPV6 |
| 189 | NC_023860_TJ_China_PPV6_2012 | PPV6 |
| 190 | KX273435_U18-9_Poland_PPV6_2014 | PPV6 |
| 191 | KX384823_P15-1_Poland_PPV6_2016 | PPV6 |
| 192 | KR709263_KSU2-AZ-2014_USA_PPV6_2014 | PPV6 |
| 193 | KR709267_KSU6-IA-2014_USA_PPV6_2014 | PPV6 |
| 194 | KF999681_BJ_China_PPV6_2013 | PPV6 |
| 195 | KF999684_SC_China_PPV6_2012 | PPV6 |
| 196 | KF999682_BJ2_China_PPV6_2012 | PPV6 |
| 197 | KF999683_JS_China_PPV6_2012 | PPV6 |
| 198 | MG696112_FJLY2017_China_PPV7_2017 | PPV7 |
| 199 | KY996758_GD-2014-3_China_PPV7_2014 | PPV7 |
| 200 | KY996757_GD-2014-2_China_PPV7_2014 | PPV7 |
| 201 | MG696111_FJFZ2017_China_PPV7_2017 | PPV7 |
| **202** | **MH817777_N133_Korea_PPV7_2018** | **PPV7** |
| **203** | **MH817776_N141_Korea_PPV7_2018** | **PPV7** |
| 204 | KY996756_GD-2014-1_China_PPV7_2014 | PPV7 |
| 205 | KU563733_42_USA_PPV7_2015 | PPV7 |
| 206 | MG543460_GX28_China_PPV7_2015 | PPV7 |
| 207 | MG543464_GX32_China_PPV7_2015 | PPV7 |
| 208 | MG543463_GX31_China_PPV7_2015 | PPV7 |
| 209 | MG543459_GX6_China_PPV7_2015 | PPV7 |
| 210 | MG543457_GX3_China_PPV7_2015 | PPV7 |
| 211 | MG543456_GX2_China_PPV7_2015 | PPV7 |
| 212 | MG543465_GX34_China_PPV7_2015 | PPV7 |
| 213 | MG543461_GX29_China_PPV7_2015 | PPV7 |
| 214 | MG543462_GX30_China_PPV7_2015 | PPV7 |
| 215 | MG543466_GX35_China_PPV7_2015 | PPV7 |
| 216 | MG543467_GX44_China_PPV7_2015 | PPV7 |
| 217 | MG543471_GX49_China_PPV7_2015 | PPV7 |
| 218 | MG914435_PPV7_SWE20_Sweden_PPV7_2016 | PPV7 |
| 219 | MG543468_GX45_China_PPV7_2015 | PPV7 |
| 220 | MG543469_GX48_China_PPV7_2015 | PPV7 |
| 221 | MG543472_GX50_China_PPV7_2015 | PPV7 |
| 222 | MG543470_GX47_China_PPV7_2015 | PPV7 |
| 223 | MG543458_GX5_China_PPV7_2015 | PPV7 |
| 224 | MH422967_KF6_Korea_PPV7_2017 | PPV7 |
| 225 | MH422966_KF5_Korea_PPV7_2017 | PPV7 |
| 226 | MH422965_KF4_Korea_PPV7_2017 | PPV7 |
| 227 | MH422964_KF3_Korea_PPV7_2017 | PPV7 |
| 228 | MH422963_KF2_Korea_PPV7_2017 | PPV7 |
| 229 | MH422962_KF1_Korea_PPV7_2017 | PPV7 |

*Notes:*

- *Sequences generated in this study were given in bold face*
- *Korean porcine parvoviruses were shaded in gray*
