# Supplementary material for: PCR-based detection and genetic characterization of porcine parvoviruses in South Korea in 2018
Source: BMC Vet Res. 2020 Apr 15;16:113. doi: 10.1186/s12917-020-02329-z (PMC7161289; doi:10.1186/s12917-020-02329-z)
Supplement: Supplementary file 2 — Additional file 2. Maximum likelihood mapping. [file 12917_2020_2329_MOESM2_ESM.docx]

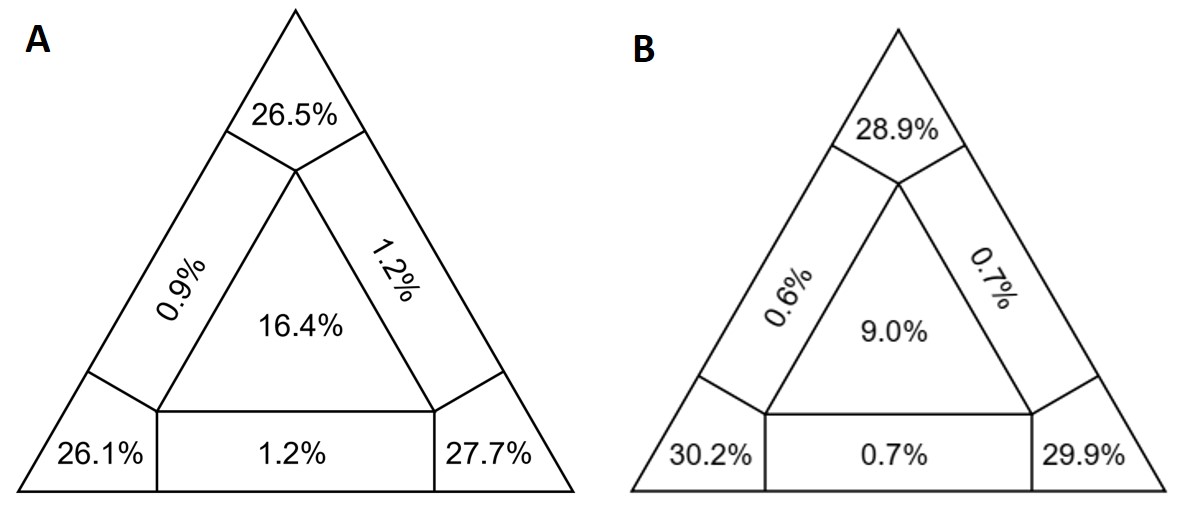


Maximum likelihood mapping of NS1 alignment from dataset 1 containing 229 sequences (including reference sequences of eight genera of subfamily *Parvovirinae*) listed in Additional file 1 (A) and dataset 2 containing sequences of dataset 1 without sequences of porcine parvovirus genotype 7 (B).
