# Supplementary figures and images for: PCR-based detection and genetic characterization of porcine parvoviruses in South Korea in 2018
Source: BMC Vet Res. 2020 Apr 15;16:113. doi: 10.1186/s12917-020-02329-z (PMC7161289; doi:10.1186/s12917-020-02329-z)

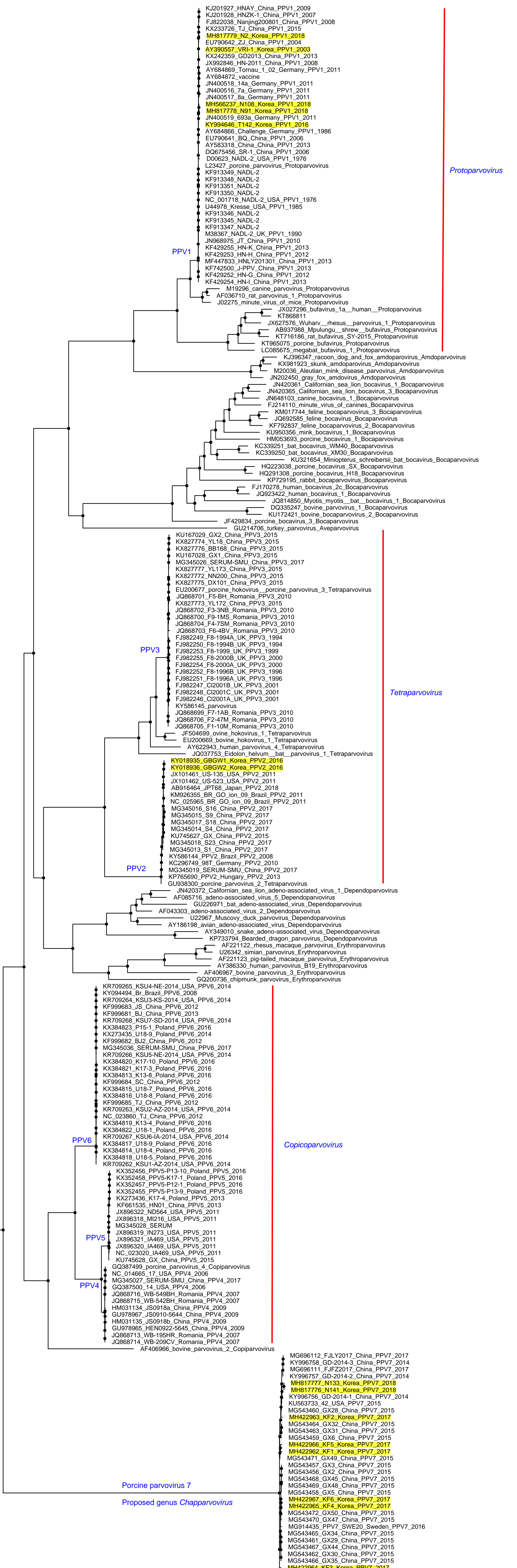

Supplement: Supplementary file 3 — Additional file 3. Phylogenetic tree of PPV1-PPV7. [file 12917_2020_2329_MOESM3_ESM.pdf]

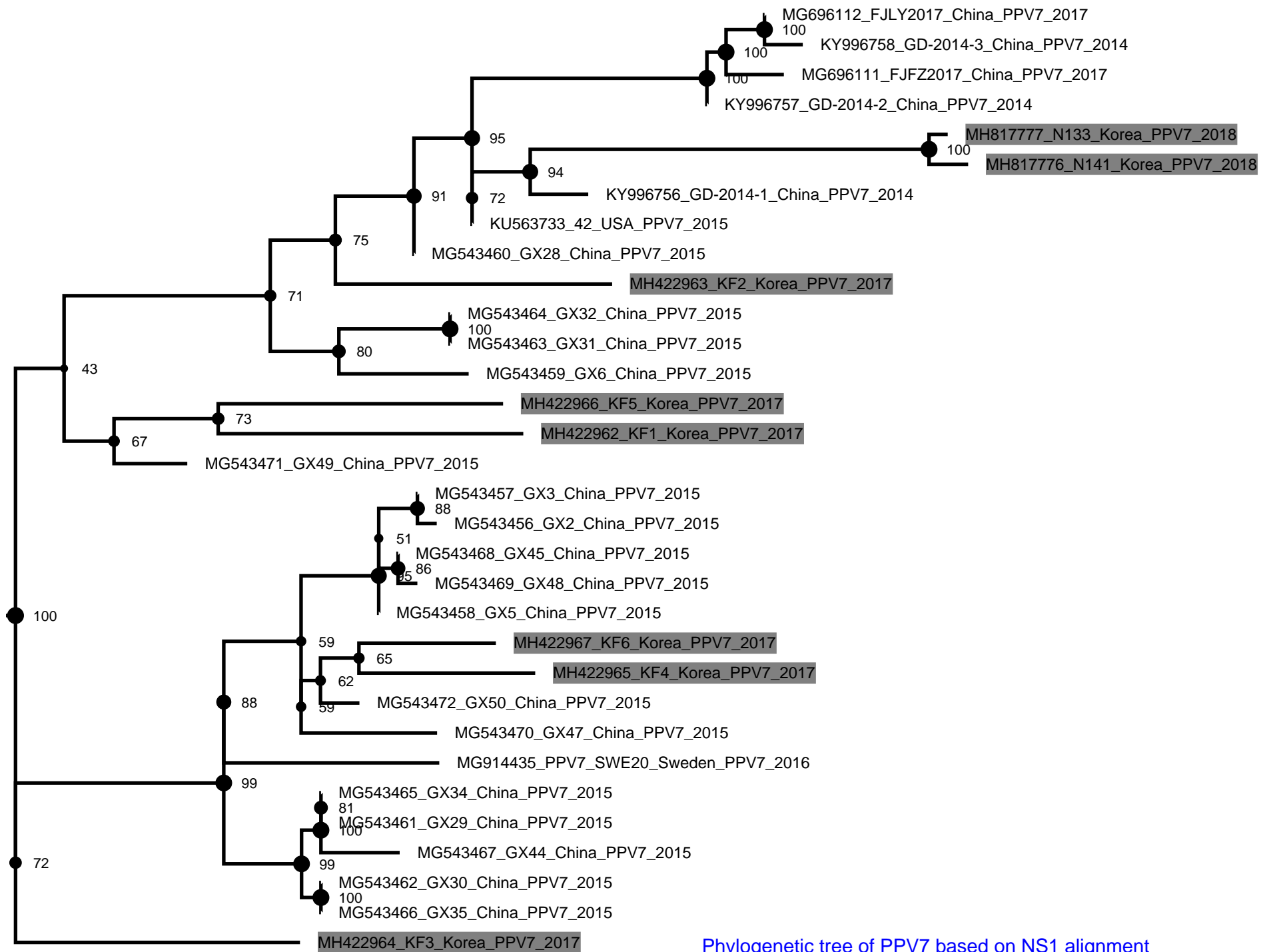

Phylogenetic tree of PPV7 based on NS1 alignment

0.009

Supplement: Supplementary file 4 — Additional file 4. Phylogenetic tree of PPV7. [file 12917_2020_2329_MOESM4_ESM.pdf]
