# Supplementary material for: PCR-based detection and genetic characterization of porcine parvoviruses in South Korea in 2018
Source: BMC Vet Res. 2020 Apr 15;16:113. doi: 10.1186/s12917-020-02329-z (PMC7161289; doi:10.1186/s12917-020-02329-z)
Supplement: Supplementary file 5 — Additional file 5. Path sampling for clock model. [file 12917_2020_2329_MOESM5_ESM.docx]

Marginal likelihood estimates for different molecular clock models of each PPV1- PPV7 dataset using path sampling

| **Dataset** | **Molecular clock model** | | | |
| --- | --- | --- | --- | --- |
|  | ***UCED*** | ***UCLD*** | ***RLC*** | ***Strict*** |
| PPV1 | -11843.2 | -11839.5 | -11682.6 | -11823.5 |
| PPV2 | -16010 | -16011.7 | -15956.7 | -16060.2 |
| PPV3 | -11313.2 | -11317.1 | -11237.4 | -11340.7 |
| PPV4 | -9463.15 | -9465.41 | -9401.27 | -9473.83 |
| PPV5 | -9450.87 | -9447.16 | -9384.3 | -9463.28 |
| PPV6 | -12364.6 | -12370.4 | -12264.5 | -12434.5 |
| PPV7 | -13015.5 | -12985 | -12915.2 | -13211.8 |

*Notes:*

- *To selecte the data best-fit molecular clock model, it was kept constant for models of* ***(i)*** *nucleotide substutition model (BEAST Model Test, implemented in BEAST 2) and* ***(ii)*** *the coalescent Bayesian skyline plot*
- *UCED: Uncorrelated exponential relaxed- clock, UCLD: Uncorrelated lognormal relaxed- clock, RLC: Random local clock, Strict: Strict molecular clock*
- *Molecular clock model resulted in the lowest marginal likelihood value (highlighted in yellow) is considered the most suitable for the corresponding data*
