# Supplementary material for: PCR-based detection and genetic characterization of porcine parvoviruses in South Korea in 2018
Source: BMC Vet Res. 2020 Apr 15;16:113. doi: 10.1186/s12917-020-02329-z (PMC7161289; doi:10.1186/s12917-020-02329-z)
Supplement: Supplementary file 6 — Additional file 6. Path sampling for coalescent model. [file 12917_2020_2329_MOESM6_ESM.docx]

Marginal likelihood estimates for different coalescent models of each PPV1- PPV7 dataset using path sampling

| **Dataset** | **Coalescent tree prior** | | |
| --- | --- | --- | --- |
|  | ***Const.*** | ***Expo.*** | ***BSP*** |
| PPV1 | -11796.1 | -11717.1 | -11682.6 |
| PPV2 | -15943.3 | -15923.9 | -15956.7 |
| PPV3 | -11235.5 | -11239.9 | -11237.4 |
| PPV4 | -9397.97 | -9400.05 | -9401.27 |
| PPV5 | -9382.05 | -9383.5 | -9384.3 |
| PPV6 | -12267.4 | -12287.7 | -12264.5 |
| PPV7 | -12855.7 | -12862 | -12915.2 |

*Notes:*

- *To selecte the data best-fit coalescent tree prior, it was kept constant for models of* ***(i)*** *nucleotide substutition model (BEAST Model Test, implemented in BEAST 2) and* ***(ii)*** *the best fit molecular clock model (additional file Table S2)*
- *This study tested 3 available coalescent models implemented in BEAST 2. The extended Bayesian skyline applied for multilocus genetic data was not applicable for genome analysis*
- *Const: coalescent constant population, Expo: coalescent exponential population, BSP: coalescent Bayesian skyline plot*
- *Coalescent model resulted in the lowest marginal likelihood value (highlighted in yellow) is considered the most suitable for the corresponding data*
