# Supplementary material for: PCR-based detection and genetic characterization of porcine parvoviruses in South Korea in 2018
Source: BMC Vet Res. 2020 Apr 15;16:113. doi: 10.1186/s12917-020-02329-z (PMC7161289; doi:10.1186/s12917-020-02329-z)
Supplement: Supplementary file 7 — Additional file 7. Details of substitution rates. [file 12917_2020_2329_MOESM7_ESM.docx]

Nucleotide substitution rates of PPV1- PPV7 inferred from the data best-fit models of molecular clock and coalescent tree prior

| **Nucleotide substitution rate** | **Protoparvovirus** | **Tetraparvovirus** | | **Copiparvovirus** | | | **Chapparvovirus** |
| --- | --- | --- | --- | --- | --- | --- | --- |
|  | ***PPV1*** | ***PPV2*** | ***PPV3*** | ***PPV4*** | ***PPV5*** | ***PPV6*** | ***PPV7*** |
| Geometric mean | 6.22E-05 | 1.35E-04 | 8.16E-04 | 4.70E-04 | 6.95E-05 | 4.90E-04 | 4.85E-03 |
| 95% HPD lower | 3.30E-05 | 4.79E-06 | 6.51E-04 | 1.51E-04 | 1.85E-08 | 2.17E-04 | 2.60E-03 |
| 95% HPD upper | 9.81E-05 | 3.96E-04 | 1.01E-03 | 1.09E-03 | 3.62E-04 | 8.31E-04 | 7.23E-03 |

*Note: Highest Posterior Density (HPD)*
