# Supplementary material for: PCR-based detection and genetic characterization of porcine parvoviruses in South Korea in 2018
Source: BMC Vet Res. 2020 Apr 15;16:113. doi: 10.1186/s12917-020-02329-z (PMC7161289; doi:10.1186/s12917-020-02329-z)
Supplement: Supplementary file 8 — Additional file 8. List of genomic sequences. [file 12917_2020_2329_MOESM8_ESM.docx]

List of 165 genomic sequences of porcine parvoviruses used in the study

| **No.** | **GenBan** | **Name of isolates** | **Country** | **Collection year** | **Genotype** |
| --- | --- | --- | --- | --- | --- |
| 1 | KF429254 | HN-I | China | 2013 | PPV1 |
| 2 | KX242359 | GD2013 | China | 2013 | PPV1 |
| 3 | KF429255 | HN-K | China | 2013 | PPV1 |
| 4 | AY583318 | China | China | 2013 | PPV1 |
| 5 | JN968975 | JT | China | 2010 | PPV1 |
| 6 | M38367 | NADL-2 | UK | 1990 | PPV1 |
| 7 | NC_001718 | NADL-2 | USA | 1976 | PPV1 |
| 8 | KF913349 | NADL-2-M13 | Hungary | 2012 | PPV1 |
| 9 | KF913347 | NADL-2-M3 | Hungary | 2012 | PPV1 |
| 10 | KF913350 | NADL-2-M23 | Hungary | 2012 | PPV1 |
| 11 | KF913351 | NADL-2-M123 | Hungary | 2012 | PPV1 |
| 12 | KF913348 | NADL-2-M12 | Hungary | 2012 | PPV1 |
| 13 | KF913346 | NADL-2-M2 | Hungary | 2012 | PPV1 |
| 14 | KF913345 | NADL-2-M1 | Hungary | 2012 | PPV1 |
| 15 | KF742500 | J-PPV | China | 2013 | PPV1 |
| 16 | KF429252 | HN-G | China | 2012 | PPV1 |
| 17 | DQ675456 | SR-1 | China | 2006 | PPV1 |
| 18 | KF429253 | HN-H | China | 2012 | PPV1 |
| 19 | D00623 | NADL-2 | USA | 1976 | PPV1 |
| 20 | JX992846 | HN-2011 | China | 2008 | PPV1 |
| 21 | MF447833 | HNLY201301 | China | 2013 | PPV1 |
| 22 | U44978 | Kresse | USA | 1985 | PPV1 |
| 23 | AY684866 | Challenge | Germany | 1986 | PPV1 |
| 24 | KY994646 | T142 | Korea | 2016 | PPV1 |
| 25 | EU790641 | BQ | China | 2006 | PPV1 |
| 26 | AY390557 | VRI-1 | Korea | 2003 | PPV1 |
| 27 | JN400516 | 7a | Germany | 2011 | PPV1 |
| 28 | JN400517 | 8a | Germany | 2011 | PPV1 |
| 29 | AY684869 | Tornau/1/02 | Germany | 2011 | PPV1 |
| 30 | AY684872 | vaccine-virus-IDT | Germany | 1964 | PPV1 |
| 31 | JN400518 | 14a | Germany | 2011 | PPV1 |
| 32 | JN400519 | 693a | Germany | 2011 | PPV1 |
| 33 | KJ201927 | HNAY | China | 2009 | PPV1 |
| 34 | KJ201928 | HNZK-1 | China | 2007 | PPV1 |
| 35 | FJ822038 | Nanjing200801 | China | 2008 | PPV1 |
| 36 | KX233726 | TJ | China | 2015 | PPV1 |
| 37 | MH566237 | N108 | Korea | 2018 | PPV1 |
| 38 | MH817778 | N91 | Korea | 2018 | PPV1 |
| 39 | MH817779 | N2 | Korea | 2018 | PPV1 |
| 40 | EU790642 | ZJ | China | 2004 | PPV1 |
| 41 | KY018935 | GBGW1 | Korea | 2016 | PPV2 |
| 42 | KY018936 | GBGW2 | Korea | 2016 | PPV2 |
| 43 | JX101461 | US-135 | USA | 2011 | PPV2 |
| 44 | KM926355 | BR/GO/ion09 | Brazil | 2011 | PPV2 |
| 45 | NC_025965 | BR/GO/ion09 | Brazil | 2011 | PPV2 |
| 46 | JX101462 | US-523 | USA | 2011 | PPV2 |
| 47 | MG345014 | S4 | China | 2016 | PPV2 |
| 48 | AB916464 | JPT68 | Japan | 2010 | PPV2 |
| 49 | MG345017 | S18 | China | 2016 | PPV2 |
| 50 | MG345016 | S16 | China | 2016 | PPV2 |
| 51 | MG345015 | S9 | China | 2016 | PPV2 |
| 52 | MG345013 | S1 | China | 2016 | PPV2 |
| 53 | MG345018 | S23 | China | 2016 | PPV2 |
| 54 | KU745627 | GX | China | 2015 | PPV2 |
| 55 | KP765690 | PPV2 | Hungary | 2013 | PPV2 |
| 56 | KY586144 | PPV2 | Brazil | 2008 | PPV2 |
| 57 | KC296749 | 98T | Germany | 2010 | PPV2 |
| 58 | MG345019 | SERUM-SMU | China | 2016 | PPV2 |
| 59 | JQ868701 | F5-BH | Romania | 2010 | PPV3 |
| 60 | JQ868703 | F6-4BV | Romania | 2010 | PPV3 |
| 61 | JQ868699 | F7-1AB | Romania | 2010 | PPV3 |
| 62 | JQ868705 | F1-10M | Romania | 2010 | PPV3 |
| 63 | JQ868706 | F2-47M | Romania | 2010 | PPV3 |
| 64 | FJ982249 | F8-1994A | UK | 1994 | PPV3 |
| 65 | FJ982250 | F8-1994B | UK | 1994 | PPV3 |
| 66 | FJ982253 | F8-1999 | UK | 1999 | PPV3 |
| 67 | FJ982255 | F8-2000B | UK | 2000 | PPV3 |
| 68 | FJ982254 | F8-2000A | UK | 2000 | PPV3 |
| 69 | FJ982251 | F8-1996A | UK | 1996 | PPV3 |
| 70 | FJ982247 | Cl2001B | UK | 2001 | PPV3 |
| 71 | FJ982248 | Cl2001C | UK | 2001 | PPV3 |
| 72 | FJ982246 | Cl2001A | UK | 2001 | PPV3 |
| 73 | FJ982252 | F8-1996B | UK | 1996 | PPV3 |
| 74 | KU167029 | GX2 | China | 2015 | PPV3 |
| 75 | KX827774 | YL18 | China | 2015 | PPV3 |
| 76 | KU167028 | GX1 | China | 2015 | PPV3 |
| 77 | KX827776 | BB168 | China | 2015 | PPV3 |
| 78 | KX827777 | YL173 | China | 2015 | PPV3 |
| 79 | MG345026 | SERUM-SMU | China | 2017 | PPV3 |
| 80 | KX827772 | NN200 | China | 2015 | PPV3 |
| 81 | KX827773 | YL172 | China | 2015 | PPV3 |
| 82 | KX827775 | DX101 | China | 2015 | PPV3 |
| 83 | JQ868702 | F3-3NB | Romania | 2010 | PPV3 |
| 84 | JQ868704 | F4-7SM | Romania | 2010 | PPV3 |
| 85 | JQ868700 | F9-1MS | Romania | 2010 | PPV3 |
| 86 | KY586145 | parvovirus-3 | Brazil | 2008 | PPV3 |
| 87 | GQ387499 | 17 | USA | 2006 | PPV4 |
| 88 | NC_014665 | 17 | USA | 2006 | PPV4 |
| 89 | MG345027 | SERUM-SMU | China | 2017 | PPV4 |
| 90 | GQ387500 | 14 | USA | 2006 | PPV4 |
| 91 | JQ868715 | WB-542BH | Romania | 2007 | PPV4 |
| 92 | JQ868716 | WB-549BH | Romania | 2007 | PPV4 |
| 93 | HM031134 | JS0918a | China | 2009 | PPV4 |
| 94 | HM031135 | JS0918b | China | 2009 | PPV4 |
| 95 | GU978967 | JS0910-5644 | China | 2009 | PPV4 |
| 96 | GU978965 | HEN0922-5645 | China | 2009 | PPV4 |
| 97 | JQ868713 | WB-195HR | Romania | 2007 | PPV4 |
| 98 | JQ868714 | WB-209CV | Romania | 2007 | PPV4 |
| 99 | KX352455 | PPV5-P13-9 | Poland | 2016 | PPV5 |
| 100 | KX352456 | PPV5-P13-10 | Poland | 2016 | PPV5 |
| 101 | KX352457 | PPV5-P12-1 | Poland | 2016 | PPV5 |
| 102 | KX273436 | K17-4 | Poland | 2013 | PPV5 |
| 103 | KX352458 | PPV5-K17-1 | Poland | 2016 | PPV5 |
| 104 | KF661535 | HN01 | China | 2013 | PPV5 |
| 105 | JX896322 | ND564 | USA | 2011 | PPV5 |
| 106 | JX896319 | IN273 | USA | 2011 | PPV5 |
| 107 | JX896318 | MI216 | USA | 2011 | PPV5 |
| 108 | KU745628 | GX | China | 2015 | PPV5 |
| 109 | JX896321 | IA469 | USA | 2011 | PPV5 |
| 110 | NC_023020 | IA469 | USA | 2011 | PPV5 |
| 111 | JX896320 | IA469 | USA | 2011 | PPV5 |
| 112 | MG345028 | SERUM-SMU | China | 2017 | PPV5 |
| 113 | KX384815 | U18-7 | Poland | 2016 | PPV6 |
| 114 | KX384816 | U18-8 | Poland | 2016 | PPV6 |
| 115 | KX384817 | U18-9 | Poland | 2016 | PPV6 |
| 116 | KX384822 | U18-1 | Poland | 2016 | PPV6 |
| 117 | KX384814 | U18-4 | Poland | 2016 | PPV6 |
| 118 | KX384818 | U18-5 | Poland | 2016 | PPV6 |
| 119 | KX384819 | K13-4 | Poland | 2016 | PPV6 |
| 120 | KR709265 | KSU4-NE-2014 | USA | 2014 | PPV6 |
| 121 | KR709266 | KSU5-NE-2014 | USA | 2014 | PPV6 |
| 122 | KR709268 | KSU7-SD-2014 | USA | 2014 | PPV6 |
| 123 | KX384820 | K17-10 | Poland | 2016 | PPV6 |
| 124 | KX384821 | K17-3 | Poland | 2016 | PPV6 |
| 125 | KX384813 | K13-8 | Poland | 2016 | PPV6 |
| 126 | KY094494 | Br | Brazil | 2008 | PPV6 |
| 127 | KR709262 | KSU1-AZ-2014 | USA | 2014 | PPV6 |
| 128 | KR709264 | KSU3-KS-2014 | USA | 2014 | PPV6 |
| 129 | MG345036 | SERUM-SMU | China | 2017 | PPV6 |
| 130 | KF999685 | TJ | China | 2012 | PPV6 |
| 131 | NC_023860 | TJ | China | 2012 | PPV6 |
| 132 | KX273435 | U18-9 | Poland | 2014 | PPV6 |
| 133 | KX384823 | P15-1 | Poland | 2016 | PPV6 |
| 134 | KR709263 | KSU2-AZ-2014 | USA | 2014 | PPV6 |
| 135 | KR709267 | KSU6-IA-2014 | USA | 2014 | PPV6 |
| 136 | KF999681 | BJ | China | 2013 | PPV6 |
| 137 | KF999684 | SC | China | 2012 | PPV6 |
| 138 | KF999682 | BJ2 | China | 2012 | PPV6 |
| 139 | KF999683 | JS | China | 2012 | PPV6 |
| 140 | MG696112 | FJLY2017 | China | 2017 | PPV7 |
| 141 | KY996758 | GD-2014-3 | China | 2014 | PPV7 |
| 142 | KY996757 | GD-2014-2 | China | 2014 | PPV7 |
| 143 | MG696111 | FJFZ2017 | China | 2017 | PPV7 |
| 144 | MH817777 | N133 | Korea | 2018 | PPV7 |
| 145 | MH817776 | N141 | Korea | 2018 | PPV7 |
| 146 | KY996756 | GD-2014-1 | China | 2014 | PPV7 |
| 147 | KU563733 | 42 | USA | 2015 | PPV7 |
| 148 | MG543460 | GX28 | China | 2015 | PPV7 |
| 149 | MG543464 | GX32 | China | 2015 | PPV7 |
| 150 | MG543463 | GX31 | China | 2015 | PPV7 |
| 151 | MG543459 | GX6 | China | 2015 | PPV7 |
| 152 | MG543457 | GX3 | China | 2015 | PPV7 |
| 153 | MG543456 | GX2 | China | 2015 | PPV7 |
| 154 | MG543465 | GX34 | China | 2015 | PPV7 |
| 155 | MG543461 | GX29 | China | 2015 | PPV7 |
| 156 | MG543462 | GX30 | China | 2015 | PPV7 |
| 157 | MG543466 | GX35 | China | 2015 | PPV7 |
| 158 | MG543467 | GX44 | China | 2015 | PPV7 |
| 159 | MG543471 | GX49 | China | 2015 | PPV7 |
| 160 | MG914435 | SWE20 | Sweden | 2016 | PPV7 |
| 161 | MG543468 | GX45 | China | 2015 | PPV7 |
| 162 | MG543469 | GX48 | China | 2015 | PPV7 |
| 163 | MG543472 | GX50 | China | 2015 | PPV7 |
| 164 | MG543470 | GX47 | China | 2015 | PPV7 |
| 165 | MG543458 | GX5 | China | 2015 | PPV7 |
