# Supplementary material for: PCR-based detection and genetic characterization of porcine parvoviruses in South Korea in 2018
Source: BMC Vet Res. 2020 Apr 15;16:113. doi: 10.1186/s12917-020-02329-z (PMC7161289; doi:10.1186/s12917-020-02329-z)
Supplement: Supplementary file 10 — Additional file 10. Posterior distribution of substitution models. [file 12917_2020_2329_MOESM10_ESM.docx]

Posterior distribution of substitution models. The figures were produced by BModelAnalyser application within BEAST2 package. The area of the circle surrounding each model is proportional to the the posterior support for that model. Blue circles indicate models are inside the 95% credible set, models with red circles are outside of this set, and models without circles have negligible support. In all PP1- PPV7 datasets, there is no single preferred substitution model as the posterior probability is spread over a number of alternative substitution models. By specifying bModelTest, nucleotide substitution model can be inferred during the MCMC analysis and does not need to be pre-determined. Unnamed models are given in the form of six digit codes.
